# Super‐Enhancer‐Driven IRF2BP2 is Activated by Master Transcription Factors and Sustains T‐ALL Cell Growth and Survival

**DOI:** 10.1002/advs.202407113

**Published:** 2024-10-25

**Authors:** Juanjuan Yu, Zimu Zhang, Yanling Chen, Jianwei Wang, Gen Li, Yanfang Tao, Yongping Zhang, Yang Yang, Chenyue Zhang, Tiandan Li, Jia Cheng, Tongtign Ji, Zhongling Wei, Wenjuan Wang, Fang Fang, Wei Jiang, Peipei Chu, Hongli Yin, Di Wu, Xiaolu Li, Xiaodong Wang, Jun‐Jie Fan, Shaoyan Hu, Zhen‐Hong Zhu, Shuiyan Wu, Jun Lu, Jian Pan

**Affiliations:** ^1^ Children's Hospital of Soochow University Suzhou 215003 China; ^2^ Institute of Pediatric Research Children's Hospital of Soochow University Suzhou Jiangsu 215003 China; ^3^ Department of Traditional Chinese Medicine Children's Hospital of Soochow University Suzhou Jiangsu 215003 China; ^4^ Department of Hematology Children's Hospital of Soochow University Suzhou Jiangsu 215003 China; ^5^ Department of Pharmacy Children's Hospital of Soochow University Suzhou Jiangsu 215025 China; ^6^ Department of Pediatrics Taizhou Municipal Hospital No. 581 Shifu Road Tai zhou Zhejiang 318000 China; ^7^ Department of Pediatrics Suzhou Wujiang District Children Hospital No.176 Garden Road Suzhou Jiangsu 215200 China; ^8^ Department of Orthopaedics Children's Hospital of Soochow University Suzhou Jiangsu 215003 China; ^9^ Burn and Plastic Surgery Children's Hospital of Soochow University Suzhou Jiangsu 215003 China; ^10^ Pediatric Intensive Care Unit Children's Hospital of Soochow University Suzhou Jiangsu 215003 China

**Keywords:** IRF2BP2, master TFs, RAG1, super‐enhancer, T‐ALL

## Abstract

Super enhancers (SEs) are large clusters of transcriptional enhancers driving the expression of genes crucial for defining cell identity. In cancer, tumor‐specific SEs activate key oncogenes, leading to tumorigenesis. Identifying SE‐driven oncogenes in tumors and understanding their functional mechanisms is of significant importance. In this study, a previously unreported SE region is identified in T‐cell acute lymphoblastic leukemia (T‐ALL) patient samples and cell lines. This SE activates the expression of interferon regulatory factor 2 binding protein 2 (IRF2BP2) and is regulated by T‐ALL master transcription factors (TFs) such as ETS transcription factor ERG (ERG), E74 like ETS transcription factor 1 (ELF1), and ETS proto‐oncogene 1, transcription factor (ETS1). Hematopoietic system‐specific IRF2BP2 conditional knockout mice is generated and showed that IRF2BP2 has minimal impact on normal T cell development. However, in vitro and in vivo experiments demonstrated that IRF2BP2 is crucial for T‐ALL cell growth and survival. Loss of IRF2BP2 affects the MYC and E2F pathways in T‐ALL cells. Cleavage under targets and tagmentation (CUT&Tag) assays and immunoprecipitation revealed that IRF2BP2 cooperates with the master TFs of T‐ALL cells, targeting the enhancer of the T‐ALL susceptibility gene recombination activating 1 (RAG1) and modulating its expression. These findings provide new insights into the regulatory network within T‐ALL cells, identifying potential new targets for therapeutic intervention.

## Graphical abstract

IRF2BP2 is crucial for the growth and survival of T‐cell acute lymphoblastic leukemia (T‐ALL) cells. IRF2BP2 is driven by a super‐enhancer and activated by master transcription factors, including RUNX1, ERG, and ELF1. IRF2BP2 cooperates with these transcription factors to target the enhancer of the T‐ALL susceptibility gene RAG1 and modulate its expression.

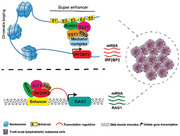

## 1 Introduction

T‐cell acute lymphoblastic leukemia (T‐ALL) is an aggressive blood cancer characterized by the malignant proliferation of T‐cell progenitors, accounting for 10% to 15% of pediatric acute leukemia.^[^ ^1^, ^2^ ^]^ While intensified treatments and bone marrow transplantation have improved survival rates, ≈20% of T‐ALL patients suffer from refractory or relapsed disease with poor outcomes and low survival rates.^[^ ^3^ ^]^ Therefore, it is imperative to further understand the biological characteristics and pathogenesis of T‐ALL to develop more effective therapies.

The Notch‐Myc signaling axis, along with transcription factors (TFs) LYL1, basic helix‐loop‐helix family member (LYL1), LMO1/2, TLX1/3, NKX2‐1, and TAL1/2, collaboratively regulate the expression of genes involved in T cell development such as the RAG family members recombination activating 1 (RAG1) and recombination activating 2 (RAG2).^[^ ^4^, ^5^, ^6^ ^]^ Genetic abnormalities in these factors have been reported to be linked to the occurrence of pediatric T‐ALL. Dysregulation of Notch signaling is found in 50% of T‐ALL cases. As a master TF in T‐ALL, overexpression of NOTCH inhibits p53‐mediated apoptosis.^[^ ^7^ ^]^ Studies have shown that in T‐ALL cells, overexpression of TAL1 leads to elevated expression levels of several TFs, including RUNX family transcription factor 1 (RUNX1), MYB proto‐oncogene, transcription factor (MYB), ETS transcription factor ERG (ERG), and ETS proto‐oncogene 1, transcription factor (ETS1), thereby promoting leukemogenesis.^[^ ^8^, ^9^, ^10^, ^11^, ^12^, ^13^ ^]^

Super‐enhancers (SEs) are clusters of enhancers where the levels of TFs, cofactors, and epigenetic modifications are significantly higher than in typical enhancers. The landscape of SEs varies greatly across different cell types.^[^ ^14^ ^]^ A set of master TFs form an interconnected core transcriptional regulatory circuit (CRC) by directly co‐occupying both their own super‐enhancers and each other's, driving gene expression that determines cell identity and cell type‐specific functions.^[^ ^15^, ^16^ ^]^ In tumors, SEs create a “locked” regulatory state, leading to uncontrolled proliferation of cancer cells.^[^ ^17^ ^]^ Histone modifications such as histone H3 lysine 27 acety (H3K27ac) and monomethylation of histone H3 at lysine 4 (H3K4me1) are commonly used to identify SEs, aiding in the identification of key oncogenes in tumors and providing significant value in understanding their pathogenic mechanisms.^[^ ^14^ ^]^ RUNX1, as one of the key members of the CRC of T‐ALL, has been validated for its crucial function in previous studies.^[^ ^13^ ^]^ The Cyclin dependent kinase 7 (CDK7) inhibitor THZ1 can eliminate T‐ALL malignancy by inhibiting the SE‐mediated regulation of RUNX1 expression.^[^ ^18^ ^]^ Subsequent research identified that a set of master TFs, including RUNX1, ERG, and ETS1, are involved in the construction of the SE‐mediated CRC in T‐ALL cells.^[^ ^13^ ^]^

Our previous research in esophageal adenocarcinoma identified E74 like ETS transcription factor 3 (ELF3), KLF transcription factor 5 (KLF5), GATA binding protein 6 (GATA6), and ETS homologous factor (EHF) as master TFs regulated by SEs, forming CRC that promotes tumor cell survival and proliferation.^[^ ^19^ ^]^ In neuroblastoma, we identified several key oncogenes regulated by SEs driving tumor progression. In leukemia, our prior research found that GNE‐987, a von Hippel‐Lindau‐based Proteolysis Targeting Chimera (PROTAC) targeting the critical SE reader Bromodomain containing 4 (BRD4), can inhibit the expression of a range of SE‐driven genes and hinder the survival of T‐ALL and acute myeloid leukemia (AML) cells.^[^ ^20^, ^21^, ^22^ ^]^ In this study, to identify new key oncogenes in T‐ALL, we built profiles of SEs by analyzing ChIP‐seq data from clinical samples and cell lines. We identified IRF2BP2 as an SE‐driven gene in T‐ALL, playing a vital role in maintaining T‐ALL cell survival and proliferation. IRF2BP2 cooperates with several master TFs of T‐ALL cells to stimulate the expression of the T‐ALL susceptibility gene RAG1. Our data provide new insights into the regulatory network within T‐ALL cells, offering potential new targets for therapeutic intervention.

## 2 Results

### 2.1 IRF2BP2 is a Super Enhancer‐Associated Gene in T‐ALL Cells

Seven bone marrow samples from our newly diagnosed pediatric T‐ALL patients (Tables S1 and S2, Supporting Information) and the T‐ALL cell line J.gamma1 were collected for the H3K27ac ChIP assay and super‐enhancer identification. A reanalysis of publicly available ChIP‐seq data from five T‐ALL cell lines was also performed (Table S3, Supporting Information).

**Figure 1:**
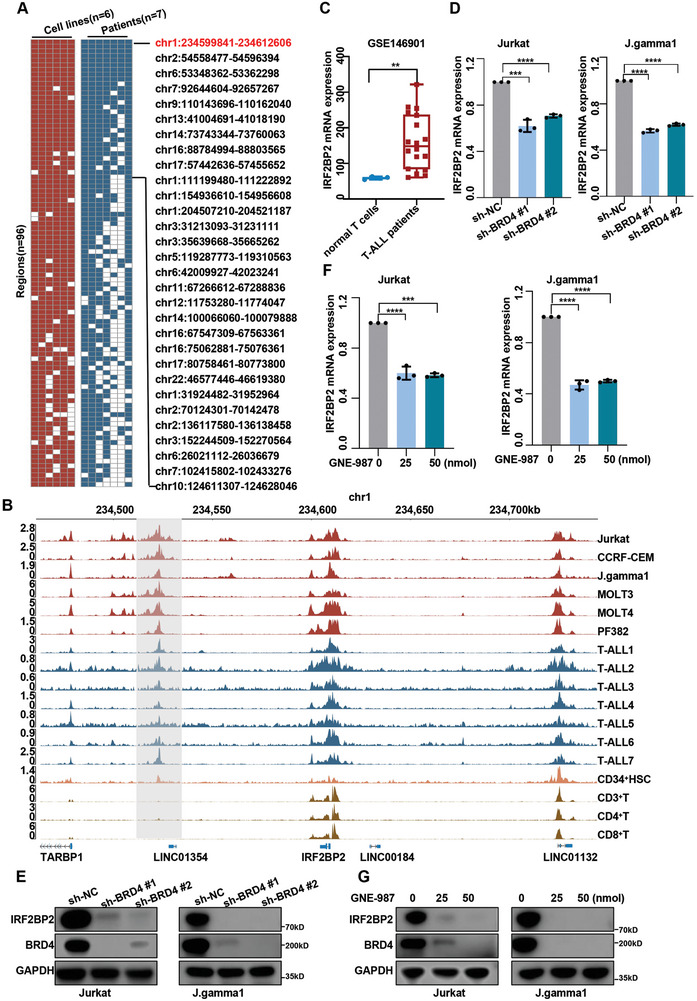
IRF2BP2 is a super enhancer‐associated gene in T‐ALL cells. A) the landscape of super‐enhancers (SEs) across these 13 samples, highlighting regions where SEs ranked in the top 60% in each sample and were present in at least 70% of the samples analyzed. B) Visualization of the ChIP‐seq data at the IRF2BP2 gene locus. C) Significant increase (*p* < 0.01) in IRF2BP2 mRNA expression in T‐ALL patient samples (*N* = 18) compared to normal T cells (*N* = 4). D,E) Knockdown of BRD4 in T‐ALL cell lines Jurkat and J.gamma1 (*N* = 3) resulted in a significant decrease in IRF2BP2 expression at both the protein and mRNA levels(*p *< 0.001). F,G) Treatment of Jurkat and J.gamma1 cell lines with the BRD4 inhibitor GNE‐987 (*N* = 3) led to comparable reductions in IRF2BP2 expression at both the protein and mRNA levels. two‐tailed unpaired *t* test in panel C, D, and F; Data are shown as mean ± s.e.m.

Our analysis revealed the landscape of super‐enhancers (SEs) across these 13 samples, identifying regions where SEs ranked in the top 60% in each sample and were present in at least 70% of the samples analyzed (**Figure** **1**A; Figure S1, Supporting Information). Among the nine SEs shared by all samples, we identified eight candidate genes of interest that were either overlapped with or flanked by these SEs: IRF2BP2, SPTBN1, ELOVL5, CDK6, PALM2AKAP2, ELF1, ELMSAN1 and MSI2 (Figure 1B; Figures S1–S4 and S5A, Supporting Information). Among these genes, CDK6, ELF1, SPTBN1, and MSI2 have been confirmed to be crucial in regulating T‐ALL progression.^[^ ^23^, ^24^, ^25^, ^26^ ^]^ Visualization of the ChIP‐seq data indicated significant enrichment of H3K27ac downstream of the IRF2BP2 gene in T‐ALL patients and cell lines, whereas these H3K27ac peaks were notably weaker or absent in CD3^+^T, CD4^+^T, and CD8^+^T cells (Figure 1B). However, the roles of IRF2BP2 in T‐ALL are not fully understood. We then analyzed gene expression profiles of T‐ALL patients from the GEO dataset (GSE146901), which revealed a significant increase in IRF2BP2 mRNA expression in T‐ALL patient samples compared to normal T cells (Figure 1C; Figure S5B, Supporting Information), and significant increase in IRF2BP2 protein levels in T‐ALL cell lines compared to normal T cells (Figure S6A, Supporting Information). BRD4, a crucial SE reader that collaborates with SEs to exert transcriptional regulation, was found to be essential for IRF2BP2 expression. Knockdown of BRD4 in T‐ALL cell lines Jurkat and J.gamma1 resulted in a significant decrease in IRF2BP2 expression at both the protein and mRNA levels (Figure 1D,E). Similarly, treatment of Jurkat and J.gamma1 cell lines with the BRD4 inhibitor GNE‐987 led to comparable reductions in IRF2BP2 expression. Collectively, IRF2BP2 is a newly identified SE‐associated gene that is highly expressed in T‐ALL cells (Figure 1F,G).

### 2.2 Master TFs Cooperatively Regulate the Super Enhancer of IRF2BP2

**Figure 2:**
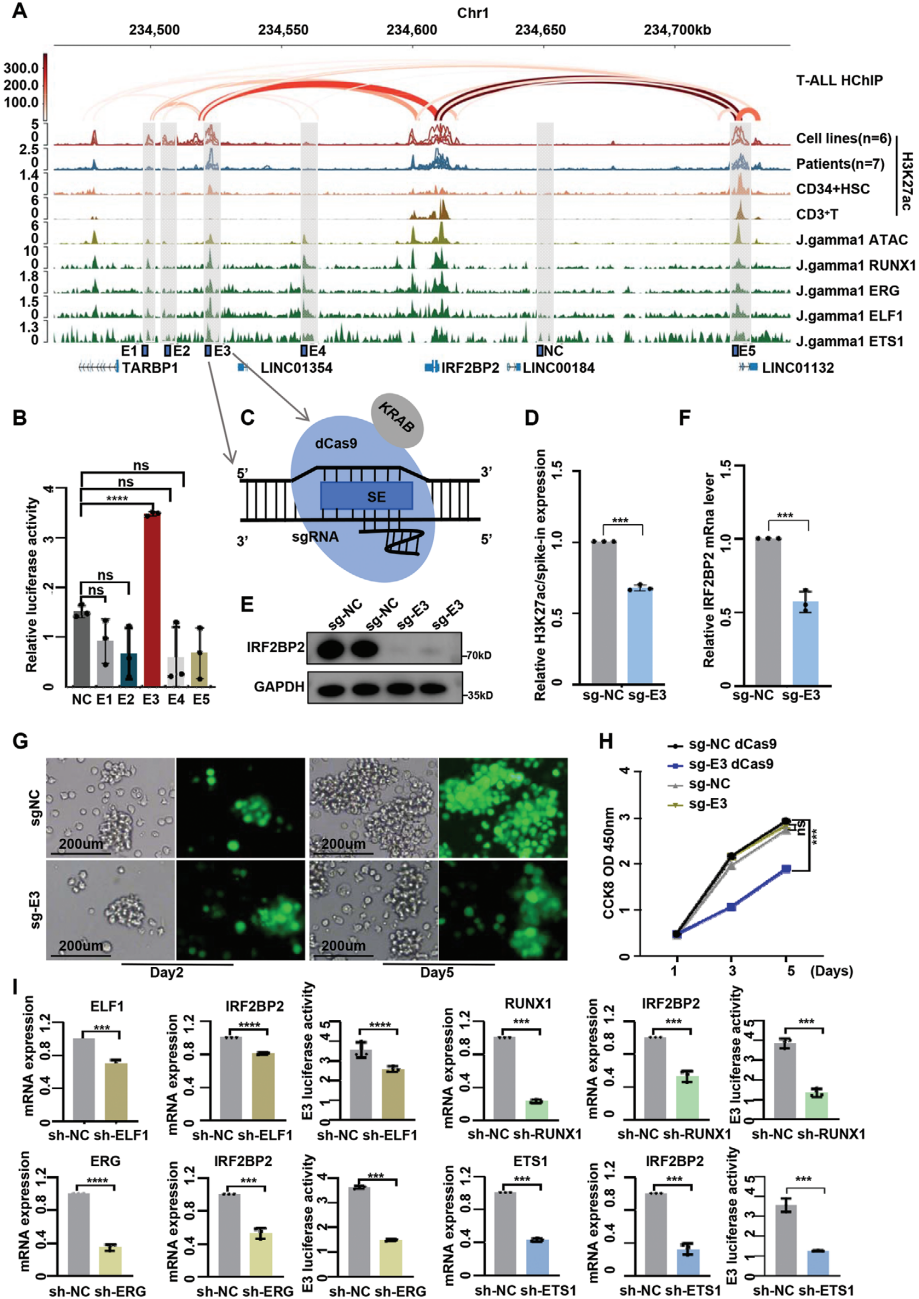
Master TFs cooperatively regulates the super enhancer of IRF2BP2. A) ChIP‐Seq profiles for H3K27Ac and CUT&Tag profiles for master TFs at IRF2BP2 super‐enhancer loci. Five candidate enhancer constituents within the SE region and one negative control were cloned into luciferase reporter vector.B) Enhancer E3 demonstrated the highest reporter activity (*N* = 3). C) Using CRISPR interference (CRISPRi) system, where sgRNA guides the dCas9/KRAB complex to suppress enhancer E3. D,E,F) Targeting E3 significantly reduced H3K27ac enrichment at this element and markedly suppressed IRF2BP2 expression at both mRNA (*N* = 3) and protein levels. G,H) Reduction in cell survival and proliferation was observed following the inhibition of enhancer E3 activity (*N* = 3). I) Interference with ELF1, RUNX1, ERG, and ETS1 significantly decreased both the reporter activity of enhancer E3 and the expression level of IRF2BP2. two‐tailed unpaired *t* test in panel B, D, Fand I; two‐way ANOVA test for analysis in H; Data are shown as mean ± s.e.m.

To further investigate the role of SE in regulating IRF2BP2 expression, we analyzed public H3K27ac HiChIP data from T‐ALL patient sample (GSE165207). The results revealed significant interactions between the SE region and the IRF2BP2 promoter. By cross‐referencing H3K27ac ChIP‐seq data and CUT&Tag of multiple master TFs (GSE267451); (Figure S7 and Tables S10–S13, Supporting Information), we identified five candidate enhancer constituents within the SE region (**Figure** **2**A). We subsequently cloned these five enhancer elements and a control region into the luciferase reporter vector. When transfected into J.gamma1 cells, enhancer E3 demonstrated the highest reporter activity (Figure 2B). To further confirm the transcriptional activation role of the enhancer element, we utilized the CRISPR interference (CRISPRi) system, where sgRNA guides the dCas9/KRAB complex to suppress enhancer E3 (Figure 2C). In J.gamma1 cells, targeting E3 significantly reduced H3K27ac enrichment at this element and markedly suppressed IRF2BP2 expression at both mRNA and protein levels (Figure 2D–F). Consistently, a reduction in cell survival and proliferation was observed following the inhibition of enhancer E3 activity (Figure 2G,H). Additionally, interference with ELF1, RUNX1, ERG, and ETS1 significantly decreased both the reporter activity of enhancer E3 and the expression level of IRF2BP2 (Figure 2I). Taken together, we validated that this key SE region, co‐occupied by master TFs, interacts with the IRF2BP2 promoter and regulates its expression in T‐ALL cells.

### 2.3 IRF2BP2 Sustains the Growth and Survival of T‐ALL Cell

**Figure 3:**
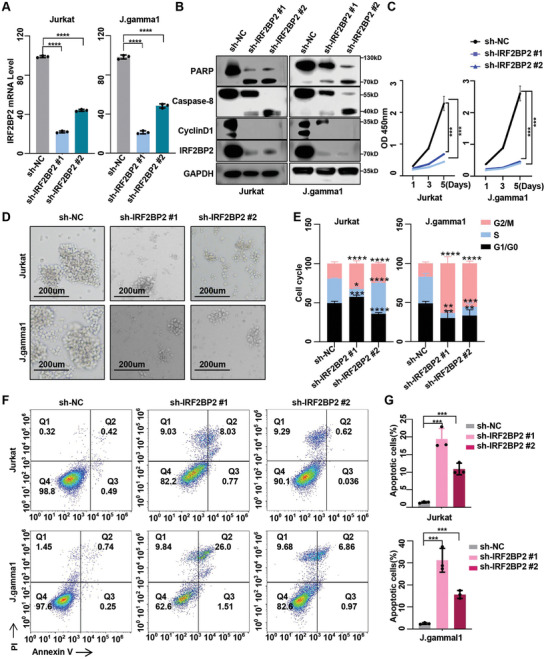
IRF2BP2 sustains the growth and survival of T‐ALL cell. A) mRNA expression of IRF2BP2 knockdown in Jurkat and J.gamma1 (*N* = 3). B) Western blot analysis indicated that IRF2BP2 knockdown decreased Cyclin D1 expression and enhanced caspase‐8 activation and PARP cleavage. C) CCK‐8 proliferation assays demonstrated that IRF2BP2 knockdown inhibited cell proliferation in T‐ALL cell lines Jurkat and J.gamma1 (*N* = 3). D) IRF2BP2‐knockdown cells exhibited membrane shrinkage, morphological changes, and an increased presence of debris in the culture medium. E) Cell cycle analysis showed that IRF2BP2 knockdown led to an extension of the G2 phase, consistent with the changes in Cyclin D1 protein levels (*N* = 3). F,G) Flow cytometry analysis revealed a significant increase in apoptosis (*N* = 3). two‐tailed unpaired *t* test in panel A, E, and G; two‐way ANOVA test for analysis in C; Data are shown as mean ± s.e.m.

To investigate the role of IRF2BP2 in T‐ALL cells, we designed shRNAs targeting two regions of IRF2BP2 and validated their knockdown efficiency using qPCR (**Figure** **3**A). Western blot analysis indicated that IRF2BP2 knockdown resulted in decreased expression of Cyclin D1 as well as enhanced caspase‐8 activation and PARP cleavage (Figure 3B). CCK‐8 proliferation assays demonstrated that IRF2BP2 knockdown inhibited cell proliferation in T‐ALL cell lines Jurkat and J.gamma1 (Figure 3C). Microscopically, IRF2BP2‐knockdown cells exhibited membrane shrinkage, morphological changes, and an increased presence of debris in the culture medium (Figure 3D). Cell cycle analysis showed that IRF2BP2 knockdown led to an extension of the G2 phase, consistent with the changes in Cyclin D1 protein levels (Figure 3E; Figure S8A, Supporting Information). Flow cytometry analysis revealed a significant increase in apoptosis (Figure 3F,G), and soft agar colony formation assays showed a reduced number of colonies in IRF2BP2‐knockdown cells compared to the control group(Figure S8B,C, Supporting Information). These functional impacts were similarly observed in Jurkat and J.gamma1 cell lines using the CRISPR/Cas9 system to knock out IRF2BP2 (Figure S9A–F, Supporting Information).

**Figure 4:**
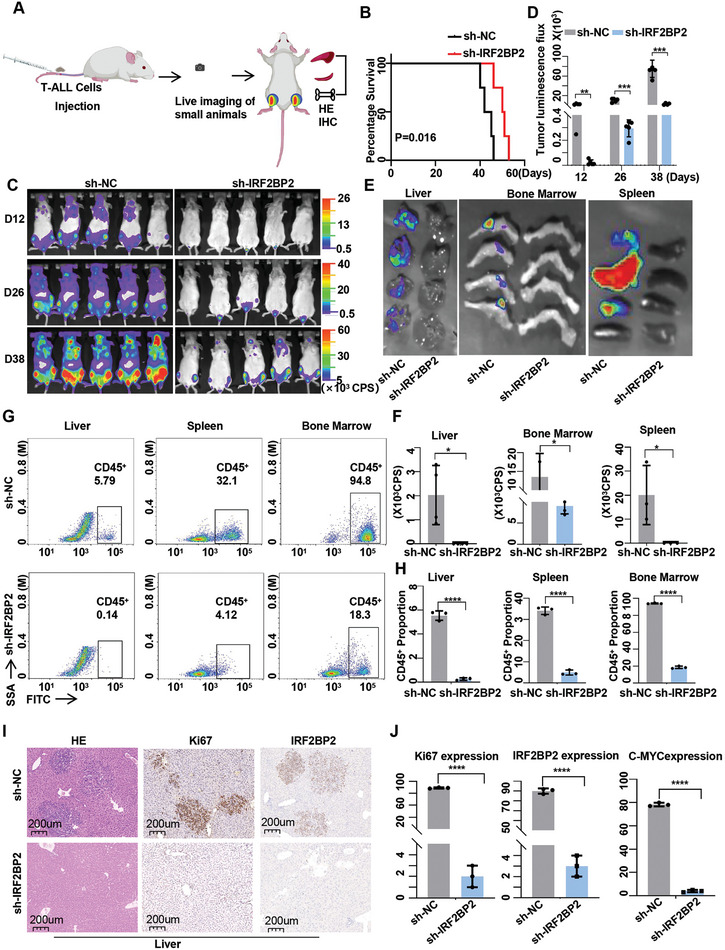
Impact of IRF2BP2 interference in vivo. A) In vivo experimental mode diagram. B) Mice injected with IRF2BP2‐knockdown cells exhibited prolonged survival compared to the control group (*N* = 8). C,D) In vivo imaging indicated significantly lower tumor cell signals in the IRF2BP2‐knockdown group (*N* = 5). E,F) Analysis of fluorescence signals (*N* = 3) in the liver, spleen, and bone marrow of sacrificed mice showed reduced tumor cell infiltration in these organs. G,H) Flow cytometry confirmed that the proportion of CD45+ cells in the liver, spleen, and bone marrow was lower in the IRF2BP2‐knockdown group compared to the control group (*N* = 3). I,J) H.E. staining and immunohistochemical analysis for Ki67 and c‐Myc demonstrated reduced tumor infiltration in the liver, spleen, and bone marrow following IRF2BP2 knockdown. long‐rank tests were conducted for overall survival analysis in panel B; two‐tailed unpaired *t* test in panel F, H, and J; Data are shown as mean ± s.e.m.

To further examine the impact of IRF2BP2 interference in vivo, IRF2BP2‐knockdown T‐ALL cells and control group were injected into NSG mice via tail vein. Mice injected with IRF2BP2‐knockdown cells exhibited prolonged survival compared to the control group (**Figure** **4**B). In vivo imaging indicated significantly lower tumor cell signals in the IRF2BP2‐knockdown group (Figure 4C,D). Analysis of fluorescence signals in the liver, spleen, and bone marrow of sacrificed mice showed reduced tumor cell infiltration in these organs (Figure 4E,F). Flow cytometry confirmed that the proportion of CD45^+^ cells in the liver, spleen, and bone marrow was lower in the IRF2BP2‐knockdown group compared to the control group (Figure 4G,H). Consistently, H.E. staining and immunohistochemical analysis for Ki67 and c‐Myc demonstrated reduced tumor infiltration in the liver, spleen, and bone marrow following IRF2BP2 knockdown (Figure 4I,J; Figure S10A–E, Supporting Information). These results collectively indicate that the growth and survival of T‐ALL cells in vitro and in vivo are dependent on IRF2BP2.

### 2.4 Normal T Cell Proportion in Mice is Unaffected by IRF2BP2

To investigate whether IRF2BP2 affects normal T cell development, a conditional knockout of IRF2BP2 was created using a Vav‐iCre transgene to selectively delete IRF2BP2 in the hematopoietic lineage (*Vav‐iCre^+/−^;Irf2bp2^fl/fl^* mice) (Figure S11A–D, Supporting Information). Western blot analysis confirmed the efficient deletion of IRF2BP2 in the bone marrow of *Vav‐iCre^+/−^;Irf2bp2^fl/fl^* mice, validating the construction of the knockout model (Figure S11E, Supporting Information).

**Figure 5:**
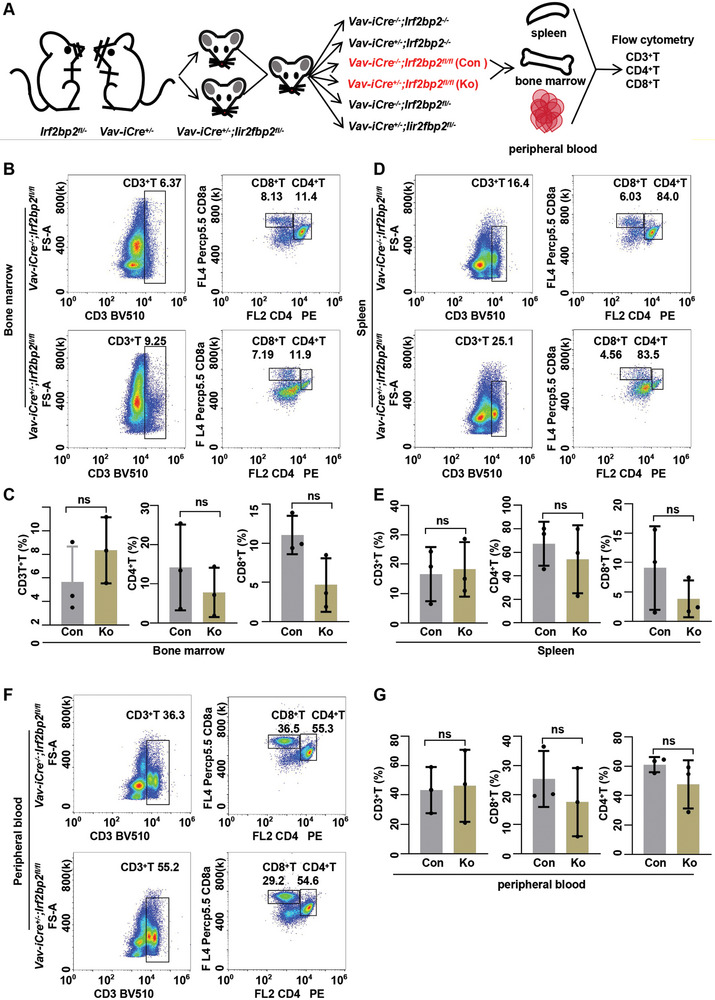
Normal T cell development in mice is unaffected by IRF2BP2. A) Pattern diagram of gene knockout mouse construction. B–G) Flow cytometry analysis revealed that the percentages of CD3^+^T, CD4^+^T, and CD8^+^T cells in the spleen, bone marrow, and peripheral blood of IRF2BP2fl/fl/Vav‐iCre± mice were similar to those observed in wild type controls two‐tailed unpaired *t* test in panel C, E, and G; Data are shown as mean ± s.e.m.

Mice were sacrificed at 6 weeks of age. Flow cytometry analysis revealed that the percentages of CD3^+^T, CD4^+^T, CD8^+^T and CD25^+^Foxp3^+^Treg cells in the spleen, bone marrow, and peripheral blood of *Vav‐iCre^+/−^;Irf2bp2^fl/fl^* mice were similar to those observed in *Vav‐iCre^−/−^;Irf2bp2^fl/fl^* controls (**Figure** **5**B–G; Figure S12, Supporting Information). These results indicate that the proportion of CD3^+^T, CD4^+^T, CD8^+^T and CD25^+^Foxp3^+^Treg cells in the normal mouse hematopoietic system does not depend on IRF2BP2.

To further investigate the effect of IRF2BP2 on normal T cells, we magnetically sorted CD3^+^ T cells from the spleens of *Vav‐iCre^+/−^;Irf2bp2^fl/fl^* mice and *Vav‐iCre^−/−^;Irf2bp2^fl/fl^* controls for RNA‐seq analysis (Figures S13A,B and S14A and Table S5 Supporting Information). GSEA analysis revealed that compared to *Vav‐iCre^−/−^;Irf2bp2^fl/fl^* controls, T cells from *Vav‐iCre^+/−^;Irf2bp2^fl/fl^* mice exhibited enhanced E2F and IL2/STAT5 signaling (Figure S14B—D and Table S6, Supporting Information).

### 2.5 IRF2BP2 Significantly Affects MYC and E2F Pathways in T‐ALL Cells

**Figure 6:**
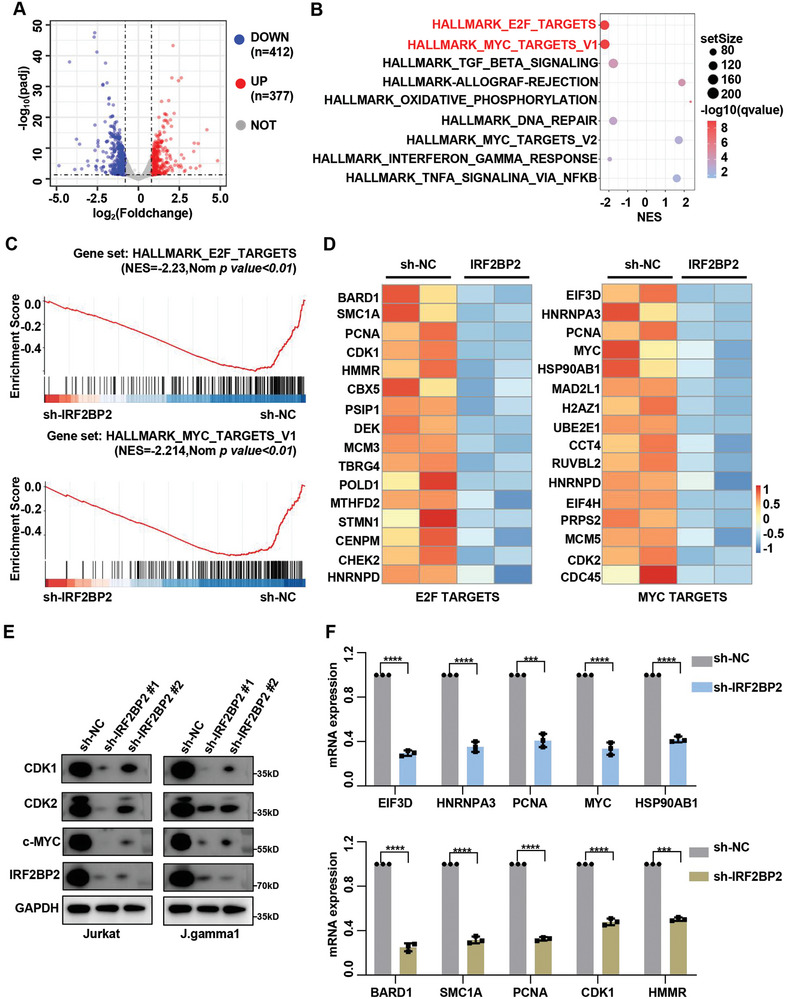
Knockdown of IRF2BP2 affects MYC and E2F pathways. A) The RNA‐seq analysis on IRF2BP2‐knockdown and control J.gamma1 cells. log2FoldChange≥0.5, FDR<0.05. B–D) Gene Set Enrichment Analysis (GSEA) using Hallmark gene sets revealed significant enrichment for E2F‐target genes and MYC‐target genes in the IRF2BP2‐knockdown group compared to controls. E) Western blot analysis further confirmed the reduction in levels of CDK1, CDK2, and c‐Myc. F) qPCR analysis revealed downregulation of several other MYC and E2F target genes, including EIF3D, HNRNPA, PCNA, HSP90AB1, BARD1, SMC1A, HMMR, and CDK1 in IRF2BP2‐knockdown cells (*N* = 3). two‐tailed unpaired *t* test in panel F; Data are shown as mean ± s.e.m.

To investigate the downstream genes influenced by IRF2BP2 in T‐ALL, we performed RNA‐seq analysis on IRF2BP2‐knockdown and control J.gamma1 cells. In the IRF2BP2‐knockdown group, 412 genes were downregulated, and 377 genes were upregulated (log2FoldChange≥0.5, FDR<0.05) (**Figure** **6**A; Table S7, Supporting Information). Gene Set Enrichment Analysis (GSEA) using Hallmark gene sets revealed significant enrichment for E2F‐target genes and MYC‐target genes in the IRF2BP2‐knockdown group compared to controls (Figure 6B–D; Figure S15 and Table S8, Supporting Information). E2F‐target genes and Myc‐target genes are crucial for the survival and proliferation of T‐ALL cells. Consistently, western blot analysis further confirmed the reduction in levels of CDK1, CDK2, and c‐Myc (Figure 6E). Additionally, qPCR analysis revealed downregulation of several other MYC and E2F target genes, including EIF3D, HNRNPA, PCNA, HSP90AB1, BARD1, SMC1A, HMMR, and CDK1 in IRF2BP2‐knockdown cells (Figure 6F).

### 2.6 IRF2BP2 Regulates T‐ALL Cell Growth and Survival Through RAG1 Enhancer Modulation

**Figure 7:**
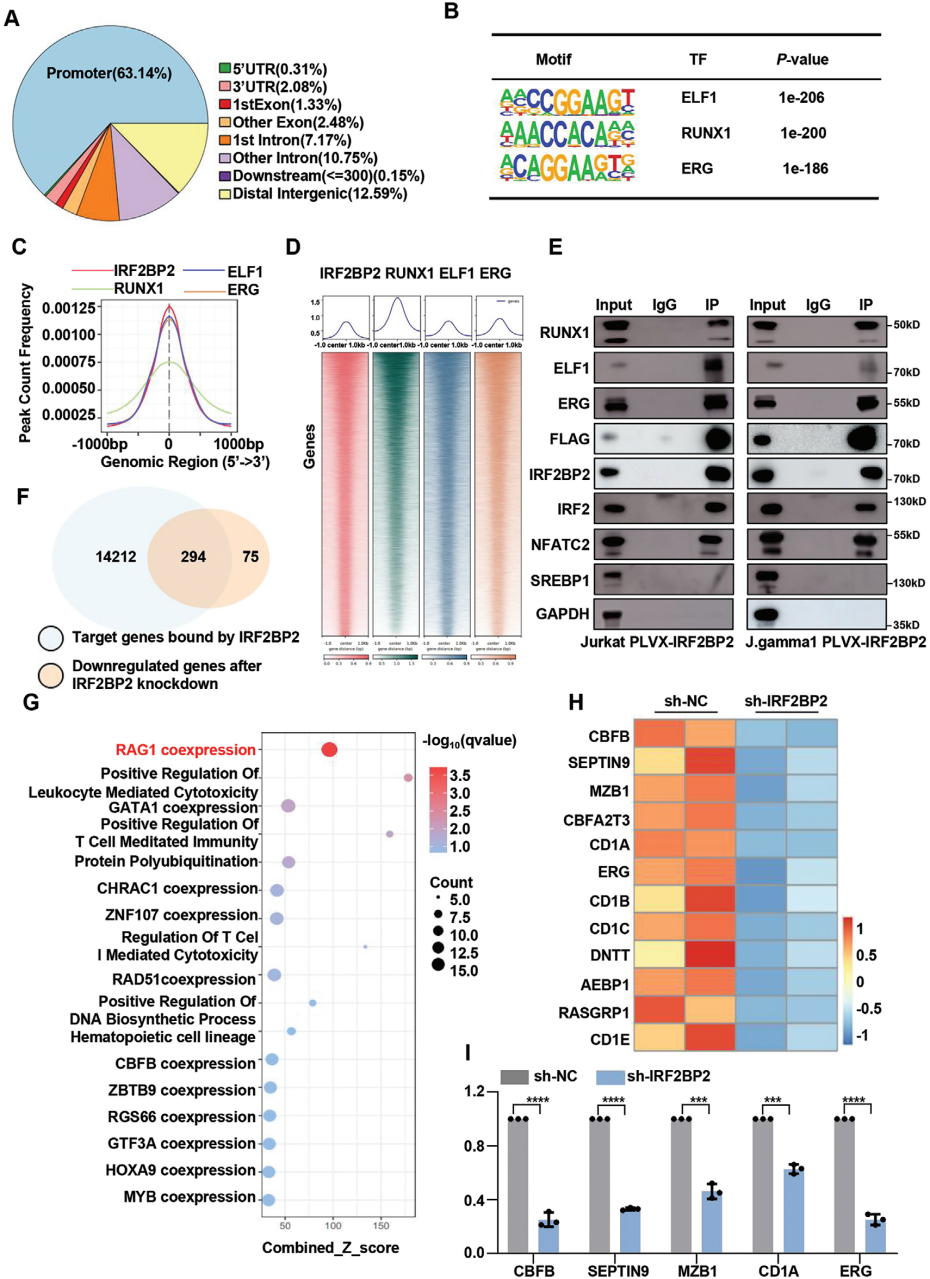
IRF2BP2 regulates T‐ALL cell growth and survival through RAG1 enhancer modulation. A) CUT&Tag analysis to determine the targets of IRF2BP2 in J.gamma1 cells, revealing ≈63% peaks found at promoters. B) Motif analysis of IRF2BP2 binding sites using Homer indicated that these peaks were enriched for the master TFs RUNX1, ERG, and ELF1. C,D) Combined analysis of CUT&Tag data for IRF2BP2, RUNX1, ERG, and ELF1 demonstrated a high correlation between IRF2BP2 occupancy and these master TFs in terms of both localization and signal intensity. E) Co‐immunoprecipitation experiments revealed the cooperation between IRF2BP2 and RUNX1, ERG, and ELF1. F) CUT&Tag analysis of IRF2BP2 identified 14506 potential target genes. Among these, 294 genes were downregulated in IRF2BP2‐knockdown cells. G,H) These 294 genes were significantly enriched in the RAG1 coexpression dataset, as assessed by EnrichR. I) qPCR analysis further confirmed the reduced expression levels of these genes such as CBFB, SEPTIN9, CBFA2T3, CD1A, and ERG in IRF2BP2‐knockdown cells (*N* = 3). Two‐tailed unpaired *t* test in panel I; Data are shown as mean ± s.e.m.

Given IRF2BP2 as an important co‐transcription factor, we conducted CUT&Tag analysis to determine the targets of IRF2BP2 in J.gamma1 cells, revealing ≈63% peaks found at promoters (**Figure** **7**A; Figure S15 and Table S10, Supporting Information). Motif analysis of IRF2BP2 binding sites using Homer indicated that these peaks were enriched for the master TFs RUNX1, ERG, and ELF1 (Figure 7B). RUNX1, ERG, and ELF1 are SE‐driven genes that are highly expressed in T‐ALL cells (Figures S1 and S17, Supporting Information). Previous studies have reported that RUNX1, ERG, and ELF1 function as master transcription factors, directly co‐occupying their own super‐enhancers as well as each other's, forming an interconnected auto‐regulatory loop and driving gene expression critical for determining cell identity and cell type‐specific functions.^[^ ^16^ ^]^ A combined analysis of CUT&Tag data for IRF2BP2, RUNX1, ERG, and ELF1 demonstrated a high correlation between IRF2BP2 occupancy and these master TFs in terms of both localization and signal intensity (Figure 7C,D). Further, co‐immunoprecipitation experiments revealed that IRF2BP2 cooperates with RUNX1, ERG, and ELF1, with previously reported IRF2BP2 partners NFAT1 and IRF2^[^ ^27^, ^28^ ^]^ serving as positive controls, and SREBP1 and GAPDH included as negative controls (Figure 7E), suggesting that IRF2BP2 functions collaboratively with these master TFs in J.gamma1 cells to regulate gene expression. The CUT&Tag analysis of IRF2BP2 identified 14506 potential target genes. Among these, 294 genes were downregulated in IRF2BP2‐knockdown cells (Figure 7F). Notably, these 294 genes were significantly enriched in the RAG1 coexpression dataset, as assessed by EnrichR (Figure 7G,H; Tables S14 and S15, Supporting Information). Within this dataset, genes such as CBFB, SEPTIN9, CBFA2T3, CD1A, and ERG have been previously reported to be closely associated with leukemia development and progression.^[^ ^29^, ^30^, ^31^, ^32^, ^33^ ^]^ qPCR analysis further confirmed the reduced expression levels of these genes in IRF2BP2‐knockdown cells (Figure 7I).

**Figure 8:**
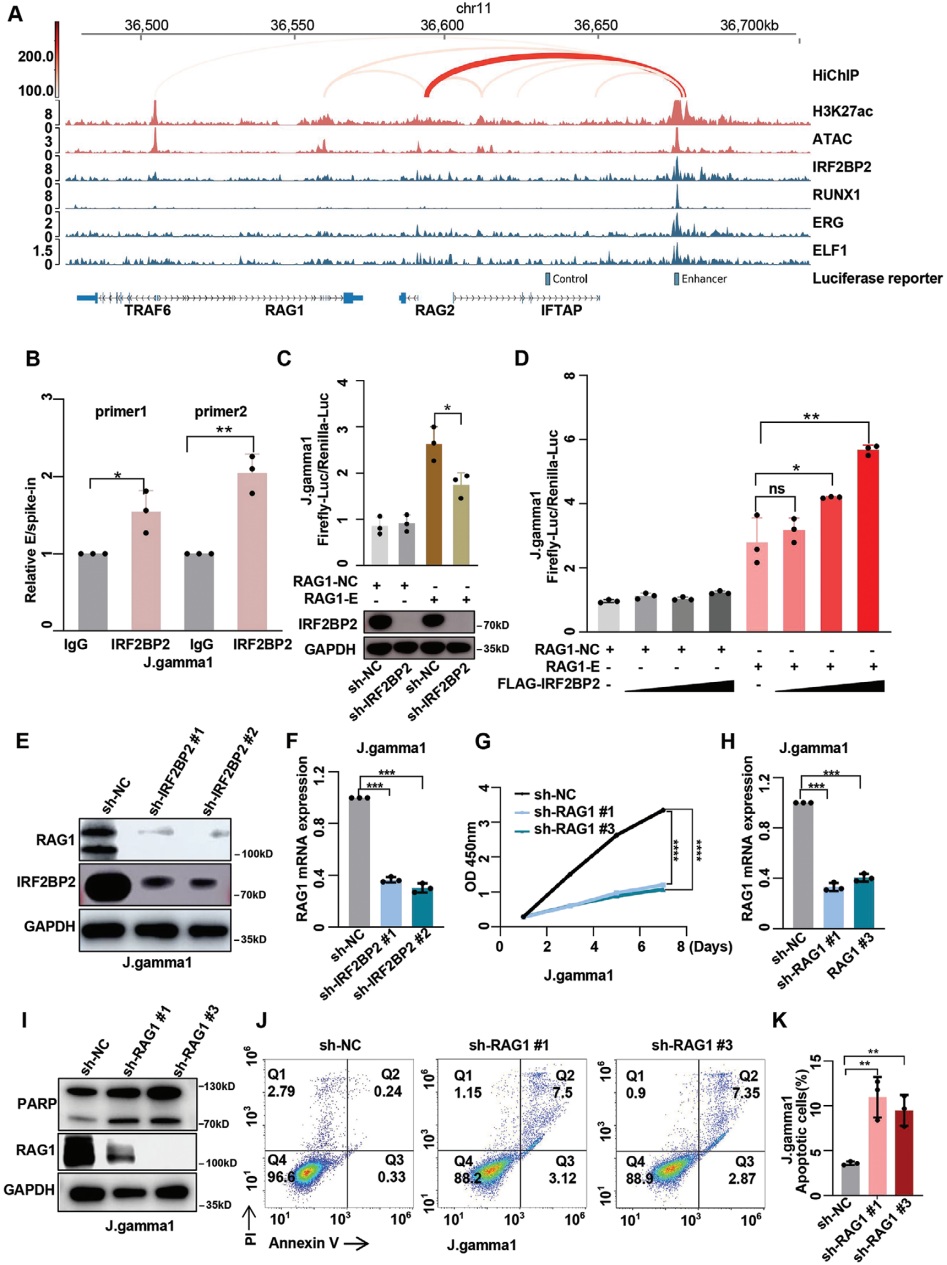
IRF2BP2 protein regulate the RAG1 gene promote T‐ALL occurrence. A). Analysis of IRF2BP2 CUT&Tag data alongside public H3K27ac HiChIP data from T‐ALL patient sample. The binding sites of IRF2BP2 on an enhancer element, co‐occupied by master transcription factors, showed significant interaction with the promoter of RAG1 gene. B) CUT&RUN qPCR (*N* = 3) using RAG1 antibody, indicating an increase in transcriptional activity of the RAG1 enhancer C) In J.gamma1 cells, IRF2BP2 knockdown resulted in the luciferase signal from RAG1 enhancer decreased (*N* = 3), D) Increased the dose of Flag‐IRF2BP2, the the luciferase signal from the RAG1 enhancer increased, indicating that RAG1 expression is dependent on IRF2BP2 (*N* = 3). E,F) Knockdown of IRF2BP2 led to decreased mRNA and protein expression of RAG1 in J.gamma1 cells (*N* = 3). G) CCK8 of knockdown RAG1 in J.gamma1 cells (N = 3), H‐I) Expression of mRNA and protein of RAG1 when knockdown RAG1 in J.gamma1 cells (*N* = 3), J‐K) Apoptosis of Knockdown of RAG1 in J.gamma1 cells (*N* = 3). two‐tailed unpaired *t* test in panel B, C, D, F, H and K; two‐way ANOVA test for analysis in G; Data are shown as mean ± s.e.m.

To investigate the relationship between IRF2BP2 protein and the RAG1 gene, we analyzed IRF2BP2 CUT&Tag data alongside public H3K27ac HiChIP data from T‐ALL patient sample (GSE165207). The binding sites of IRF2BP2 on an enhancer element, co‐occupied by master transcription factors, showed significant interaction with the promoter of RAG1 gene (**Figure** **8**A). CUT&RUN‐qPCR verified the presence of IRF2BP2 at the RAG1 enhancer (Figure 8B). To determine the regulatory role of IRF2BP2 on RAG1, we cloned the RAG1 enhancer and control region into a luciferase reporter plasmid. Compared to the control, decreased luciferase signal of the RAG1 enhancer was observed in J.gamma1 cells with IRF2BP2 knockdown (Figure 8C). When co‐transfected the luciferase reporter plasmids into J.gamma1 cells with increasing doses of IRF2BP2, we observed a dose‐dependent increase in the luciferase signal from the RAG1 enhancer, indicating that IRF2BP2 functions as an activator of this enhancer (Figure 8D). Consistently, western blotting showed that IRF2BP2 knockdown resulted in decreased RAG1 expression in Jurkat and J.gamma1 cells, (Figure 8E,F; Figure S18A,B, Supporting Information). For further validation, we designed shRNAs targeting two regions of RAG1 and confirmed their knockdown efficiency using qPCR and western blot (Figure 8H,I; Figure S18D,E, Supporting Information). Knockdown of RAG1 led to inhibited growth (Figure 8G; Figure S18C, Supporting Information) and increased apoptosis in T‐ALL cells (Figure 8J,K; Figure S18F,G, Supporting Information). Due to the adjacent genomic locations of RAG1 and RAG2, and HiChIP results indicating interactions between the IRF2BP2‐bound enhancer and RAG2 in addition to RAG1, we conducted experiments related to RAG2. These experiments revealed that RAG2 expression is impacted by IRF2BP2 knockdown. Furthermore, following RAG2 knockdown, the proliferation of J.gamma1 cells was significantly inhibited (Figure S19, Supporting Information). These findings suggest that IRF2BP2 influences T‐ALL cell growth and survival by modulating the enhancer activity of RAG1 .

## 3 Discussion

The development of tumors is closely linked to the dysregulation of growth‐associated gene expression. Gene expression is controlled by DNA elements, which can be altered by changes in enhancer activity or by direct mutations in target genes. These processes can create tumor‐specific super enhancers (SEs), which drive the expression of key oncogenes and lead to tumorigenesis.^[^ ^17^ ^]^ Identifying SE‐driven oncogenes in tumors and studying their functional mechanisms is of significant value. In this study, we identified an SE region with high frequency of occurrence in samples from 7 pediatric T‐ALL patients and 6 T‐ALL cell lines, and this SE can activate interferon regulatory factor 2 binding protein 2 (IRF2BP2).

Previous research indicates that IRF2BP2 consists of two splice variants, IRF2BP2a and IRF2BP2b, and functions as a transcriptional cofactor that interacts with the C‐terminal transcription domain of IRF2.^[^ ^34^ ^]^ IRF2BP2 plays a role in maintaining cellular homeostasis and is involved in various biological functions, including cell cycle regulation, apoptosis, angiogenesis, and immune response.^[^ ^35^, ^36^, ^37^, ^38^ ^]^ Additionally, abnormal IRF2BP2 expression has been implicated in tumors, with various fusion genes such as IRF2BP2‐RARA, IRF2BP2‐CDX2, and IRF2BP2‐NTRK identified in acute promyelocytic leukemia and solid tumors.^[^ ^39^, ^40^ ^]^ The expression of IRF2BP2 is disrupted in B‐cell lymphomas^[^ ^41^ ^]^ and hepatocellular carcinoma. Overexpression of IRF2BP2 in the liver inhibits liver cancer induced by Hippo pathway inactivation.^[^ ^42^ ^]^ IRF2BP2 was marked by an SE in AML samples, consistent with its high level of expression in AML.^[^ ^43^ ^]^ Large‐scale CRISPR library screenings have shown that AML leukemia cells depend on IRF2BP2, and its disruption leads to an imbalance in inflammatory cytokine homeostasis, ultimately causing AML cell death. Further research revealed that the anti‐inflammatory effect mediated by IRF2BP2 in AML depends on the AP‐1 heterodimer composed of ATF7 and JDP2.^[^ ^44^ ^]^ Our previous studies in neuroblastoma have shown that IRF2BP2 is driven by SE, leading to significantly elevated expression levels. IRF2BP2 regulates the expression of the key gene ALK through AP‐1, thereby influencing the growth and survival of tumor cells.^[^ ^45^ ^]^ The variation in expression and functions mentioned above may be attributed to differences in the cell types studied. The role and mechanisms of IRF2BP2 in T‐ALL remain unclear.

Our research demonstrated that knockdown of IRF2BP2 inhibited the proliferation of T‐ALL cells, induced significant apoptosis, and caused cell cycle arrest at the G1/S phase. IRF2BP2‐deficient tumor cells showed suppressed growth in NSG mice. Furthermore, to investigate whether IRF2BP2 affects normal T cell development, we generated hematopoietic system‐specific IRF2BP2 conditional knockout mice and magnetically sorted T cells from the spleens of *Vav‐iCre^+/−^;Irf2bp2^fl/fl^* mice and *Vav‐iCre^−/−^;Irf2bp2^fl/fl^* controls for RNA‐seq analysis. GSEA analysis revealed that compared to controls, T cells from *Vav‐iCre^+/−^;Irf2bp2^fl/fl^* mice exhibited enhanced E2F signaling, which may explain the previously reported inhibitory role of IRF2BP2 in T cell proliferation.^[^ ^46^ ^]^ Enhanced E2F signaling may reflect increased proliferation of T cells; however, no significant changes were observed in the proportions of CD3^+^ T, CD4^+^ T, or CD8^+^ T cells in the spleen, bone marrow, or peripheral blood, possibly due to the similar influence of IRF2BP2 on other types of hematopoietic cells. Although IL2/STAT5 signaling is involved in the generation of Treg cells, the proportion of Treg cells in *Vav‐iCre^+/−^;Irf2bp2^fl/fl^* mice showed no notable differences compared to controls. This is consistent with a previous study that reported impaired expression of the high‐affinity α‐chain of IL‐2R in IRF2BP2‐overexpressing T cells; however, under in vitro induction conditions, the percentages of induced Treg cells generated in IRF2BP2‐overexpressing cells were similar to those in control vector‐transduced cells.^[^ ^46^, ^47^ ^]^ Our study suggests that IRF2BP2 may restrict the proliferation of normal T cells but has minimal impact on their subset distribution. In contrast, IRF2BP2 expression is significantly elevated in T‐ALL cells compared to normal T cells, where it plays a critical role in supporting the growth and survival of tumor cells. CUT&Tag data analysis revealed significant enrichment of a group of master transcription factors (ERG, ELF1, ETS1, RUNX1) in IRF2BP2‐SE region. ERG and ELF1 are members of the ETS transcription factor family, with studies showing that high ERG expression correlates with poor prognosis.^[^ ^48^ ^]^ Transgenic expression of Erg induces T‐ALL in mice, and its knockdown reduces the proliferation of human MOLT4 T‐ALL cells.^[^ ^33^ ^]^ ELF1 is reported to be recruited by RNA polymerase to form the transcription elongation complex (TEC), enhancing promoter strength and transcription.^[^ ^49^, ^50^ ^]^ The activity of MYB and MYC enhancers in T‐ALL is dependent on RUNX1, with its inhibition suppressing the growth of human T‐ALL cell lines and primary samples from patients.^[^ ^51^ ^]^ Our analysis indicates that these T‐ALL master TFs binding to the SE region leads to abnormal upregulation of IRF2BP2.

Studies have reported that IRF2BP2 inhibits IL‐1ß/TNFα signaling through NF‐κB, and disruption of IRF2BP2 leads to an acute inflammatory state, resulting in AML cell death.^[^ ^43^ ^]^ Similarly, our RNA‐seq analysis showed that MYC and immune‐inflammatory pathways are significantly affected in T‐ALL cell lines with IRF2BP2 knockdown. Previous studies suggested that in macrophages, IRF2BP2 has been shown to act as a transcriptional coactivator of MEF2, exerting anti‐inflammatory effects,^[^ ^47^ ^]^ while in cardiomyocytes, IRF2BP2 has been found to interact with NFAT1, functioning as a corepressor.^[^ ^27^ ^]^ In our study, joint analysis of CUT&Tag and RNA‐seq data revealed that RAG1 and its co‐expressed gene set were significantly impacted by IRF2BP2 knockdown, which was confirmed by western blot. To verify the regulatory role of IRF2BP2 on RAG1, we cloned the RAG1 enhancer into a luciferase reporter plasmid and co‐transfected it into J.gamma1 cells with increasing doses of IRF2BP2. We observed a dose‐dependent increase in the luciferase signal from the RAG1 enhancer, indicating that IRF2BP2 functions as an activator of this enhancer. RAG1 and RAG2 proteins form a DNA recombinase initiating V(D)J recombination, generating numerous Igh variable region exons essential for immune responses against various pathogens.^[^ ^52^, ^53^ ^]^ In B‐ALL, the ETV6‐RUNX1 fusion gene induces excessive RAG recombinase activity, leading to tumor progression. Reports have indicated that RAG1/2‐mediated oncogenic splicing plays a critical role in leukemia cells, affecting genomic stability.^[^ ^54^ ^]^ The E2F pathway, which is closely associated with genomic stability, is significantly impacted in T‐ALL cells upon disruption of IRF2BP2 expression, according to our RNA‐seq data. Notably, motif analysis of our IRF2BP2 CUT&Tag results revealed a significant enrichment of master TFs RUNX1, ERG, and ELF1 at IRF2BP2 binding sites. Co‐IP further confirmed their cooperation at the protein level. This differs from the significant enrichment of AP‐1 at IRF2BP2 binding sites observed in AML and neuroblastoma.^[^ ^44^, ^45^ ^]^ We propose that in T‐ALL, IRF2BP2 collaborates with master TFs to regulate the expression of the critical susceptibility gene RAG1, reshaping the intracellular environment and driving its oncogenic potency. Our data provide new insights into the regulatory network within T‐ALL cells and suggest potential new targets for therapeutic intervention.

## 4 Experimental Section

### Cell Culture

Human cell lines (293FT, Jurkat, J.gamma1, MOLT4, CCRF‐CEM, PF382) were sourced from the National Collection of Authenticated Cell Cultures (Shanghai, China), and their identities were confirmed through short tandem repeat analysis. Cells were cultured in conditions as previously reported.^[^ ^21^ ^]^

### Human Tissue Samples, ChIP‐seq, and Super Enhancer Analysis

The human tissue study was approved by the Human Care Committee of Suzhou College (approval number: 2023CS106). All procedures followed the approved guidelines. Written informed consent was obtained from all patients' legal guardians. Bone marrow samples from newly diagnosed pediatric T‐ALL patients were collected for the ChIP assay, which was conducted following established protocols detailed in previous studies.^[^ ^22^ ^]^ In brief, cells were fixed with 1% formaldehyde and quenched with 0.125 m glycine. The cells were then lysed in an ice‐cold cell lysis buffer, and the lysates were ruptured using a 1 mL insulin needle. The precipitate obtained by centrifugation was resuspended in a shearing buffer containing a protease inhibitor cocktail and sonicated with an ultrasonic cell disruptor (M220, Covaris, Massachusetts, USA) to fragment the genomic DNA into 300—800 bp segments. The sheared chromatin was incubated overnight at 4 °C with H3K27ac antibody (ab4729, Abcam). The antibody‐chromatin complexes were then captured using Dynabeads Protein G beads (10004D, Thermo Fisher Scientific) and washed with lysis buffer and TE buffer. Subsequently, the complexes were treated with RNase (#7013, CST) and Proteinase K (AM2546, Invitrogen), and the remaining DNA fragments were purified using the QIAquick PCR Purification Kit (QIAGEN). Library preparation and sequencing were performed by BGI (Shenzhen, China). The 50 bp single‐end reads were aligned to the hg38 (Ensembl) genome using BWA‐MEM. Duplicate reads were removed using Picard tools, and peaks were called using MACS (version 3.0). Super enhancers were identified using the ROSE software.

### Knockdown, Knockout, or Overexpression of Genes

The sequences of shRNA listed in the Supporting Information were synthesized and constructed into pLKO.1 vector by IGE Biotechnology, Ltd. (Guangzhou, China). GeneChem Co., Ltd (Shanghai, China) designed and constructed sgRNA targeting the enhancer and IRF2BP2 using the CRISPR/Cas9 system, along with the vectors Lenti‐Cas9‐neo and Lenti‐sgRNA‐EGFP‐puro. The vector pLVX‐EF1α‐IRES‐puro vector (Clontech), Which carries FLAG‐tagged IRF2BP2 was constructed by General Biosystems (Anhui, China). Lentiviral packaging was performed as previously described.^[^ ^21^, ^22^ ^]^ Lentiviral medium, if necessary, was concentrated by PEG‐8000 (Beyotime, China) precipitation. Cells were transduced with lentiviral particles in the presence of polybrene (Sigma‐Aldrich). After screening with puromycin (Sigma–Aldrich), knockdown or overexpression cells were established.

### Soft Agar Colony Formation Assay

Mix 750 µL of 1.25% soft agar with 750 µL of 2 × 1640 complete medium, and layer it into a 6‐well plate. Set overnight to form the bottom layer. The next day, mix 750 µL of 0.75% soft agar with 750 µL of 2 × 1640 complete medium and 7 × 10^3^ cells. Layer this mixture on top of the solidified bottom layer. Periodically supplement with 1 × 1640 complete medium. On day 20, fix the colonies with 4% paraformaldehyde and stain them with 1×Giemsa solution. Colonies were imaged and counted.

### Animal Experiments

All animal studies were approved by the Animal Care Committee of Suzhou College (approval number: CAM‐SU‐AP#: JP‐2018‐1). Six‐week‐old female NSG mice (*n* = 6 per group) received tail vein injections of luciferase‐tagged J.gamma1 cells (4 × 10^6^ cells per mouse). For the IRF2BP2 knockdown study, J.gamma1 cells were stably transduced with shRNA targeting IRF2BP2 or a scramble control. Small animal in vivo imaging was performed regularly. Mice were sacrificed when signs of distress such as piloerection, hunching, or weight loss were observed. Liver, spleen, and bone marrow tissues were collected for H.E. staining and immunohistochemistry. Flow cytometry was conducted on isolated cells using anti‐CD45 antibodies (304008, Biolegend).

For the transgenic mouse experiments, *rf2bp2^fl/fl^* mice were generated by GemPharmatech Co., Ltd. (Nanjing, China), and Vav‐iCre mice were obtained from Cyagen Biosciences (Suzhou, China). Detailed information is provided in the Supporting Information. A conditional knockout of IRF2BP2 was created using a Vav‐iCre transgene to selectively delete IRF2BP2 in the hematopoietic lineage (*Vav‐iCre^+/−^;Irf2bp2^fl/fl^* mice). The efficiency of gene knockout in bone marrow cells was confirmed by western blotting. Flow cytometry was used to analyze T cell markers, including CD3 (100233, Biolegend), CD4 (100408, Biolegend), CD8a (100734, Biolegend), CD45 (103103, BioLegend), CD4 (552052, BD Biosciences), FOXP3 (562996, BD Biosciences), CD25T (552880, BD Biosciences) in liver, spleen, and bone marrow cells. To further investigate the effect of IRF2BP2 on normal T cells, we used Mouse CD3^+^T cell Isolution Kit (CS101‐01, Vazyme) to magnetically sorted CD3+ T cells from the spleens of *Vav‐iCre^+/−^;Irf2bp2^fl/fl^* mice and *Vav‐iCre^−/−^;Irf2bp2^fl/fl^* controls for RNA‐seq analysis. All animal experiments were approved by the Animal Care and Use Committee of Soochow University and performed in accordance with institutional guidelines.

### Dual Luciferase Reporter Assay

Two million J.gamma1 cells were mixed with plasmid DNA in 100µL of High‐Performance Electroporation Solution (BTX Press). The mixture was transferred to an Electroporation cuvette plus (BTX Press), and subjected to two 7ms pulses with an interval of 100ms and a voltage of 140V using the ECM 830 Electro Square Porator (BTX Press). After electroporation, the cells were incubated in the cuvette for 10 minutes. The cell suspension was then transferred to a 6‐well plate containing 3mL of complete 1640 medium and incubated at 37 °C in a 5% CO2 incubator for 48 h. Following incubation, cells were collected, and the Dual‐Luciferase Reporter Assay was performed using the Dual‐Luciferase Reporter Assay Kit (DL101‐01, Vazyme) according to the manufacturer's protocol.

### Co‐Immunoprecipitation

Co‐immunoprecipitation was performed as previously described.^[^ ^55^ ^]^ After eluting from Anti‐FLAG M2 Affinity Gel (A2220, Sigma–Aldrich)‐bound immunocomplexes, protein levels were determined by western blotting.

### Statistical Analysis

Statistical analysis was performed using GraphPad Prism version 8.0.2 (GraphPad Software, Inc., USA), including *t* tests, long‐rank tests that were conducted for overall survival analysis. Differences were considered statistically significant when *p* < 0.05 (^*^ *p* < 0.05; ^**^ *p* < 0.01; ^***^ *p* < 0.001; ^****^ *p* < 0.0001).

## Conflict of Interest

The authors declare no conflict of interest.

## Author Contributions

J.Y., Z.Z., Y.C., J.W., and G.L. contributed equally to this work. J.P., J.L., and S.W. designed and supervised the study. J.Y. conducted the majority of the experiments, analyzed the data, and drafted the paper. Z.Z. Helped with RNA‐seq analysis, CUT&TAG analysis and contributed to manuscript writing. Y.C. and G.L. helped with animal experiments. Y.T. and Y.Z. supervised the revision of the paper. Y.Y. and C.Z. engaged in statistical analysis of clinical sample data. T.L. and J.C. took part in animal experiments. T.J., Z.W., and W.W. offered guidance on experimental techniques. F.F., W.J., and P.C. participated in Co‐IP experiments. Z.L. and R.Z. helped with CUT&TAG experiments. H.Y., D.W., X.L., and J.‐J.F. helped with the establishment of settlement experiments. X.W., S.H., and Z.‐H.Z. supervised the revision of the paper. The final draft was read by all authors who approved it.

## Supporting information



Supporting Information

## Data Availability

The sequencing and processed data files from GSE267758 (T‐ALL1‐7 and J.Gamma1 ChIP‐seq), GSE267451 (ERG IRF2BP2 RUNX1 ETS1 ELF1 CUT&Tag), and GSE267261 (RNA‐seq of J.gamma1 shIRF2BP2 and CD3^+^ T cells from *Vav‐iCre^+/−^; Irf2bp2^fl/fl^* mice) are our newly generated datasets that have been submitted to the Gene Expression Omnibus (GEO; http://www.ncbi.nlm.nih.gov/geo/) repository. Public datasets available from the GEO database include GSE165207 (T‐ALL HiChIP), GSE59657 (Jurkat ChIP‐seq), GSE76783 (CCRF‐CEM ChIP‐seq), GSE59657 (MOLT3 ChIP‐seq), GSE79288 (MOLT4 ChIP‐seq), GSE243772 (PF382 ChIP‐seq), GSE18927 (CD3^+^T, CD4^+^T, CD8^+^T ChIP‐seq), GSE231486 (HSC ChIP‐seq). Other relevant data are available from the corresponding authors upon reasonable request.
