# Supplementary material for: Super‐Enhancer‐Driven IRF2BP2 is Activated by Master Transcription Factors and Sustains T‐ALL Cell Growth and Survival
Source: Adv Sci (Weinh). 2024 Oct 25;12(1):2407113. doi: 10.1002/advs.202407113 (PMC11714186; doi:10.1002/advs.202407113)

**Super-enhancer-driven IRF2BP2 is Activated by Master Transcription Factors and Sustains T-ALL Cell Growth and Survival**

**Supporting Information**

**Detailed Experimental Section**

**Luciferase reporter assay:** Candidate enhancer constituents identified by HiChIP and ChIP-Seq data were cloned into luciferase reporter vector pGL3-Promoter (Promega). Primers were listed in the Supporting Information. The constructs underwent sequencing verification. The vectors were transfected into the J.gamma1 cell line using Lipofectamine 3000 (Invitrogen). A Renilla luciferase control vector was co-transfected to enable normalization. Luciferase activity was assessed 48 hours post-transfection using the Dual-Luciferase Reporter Assay System (Promega).

**RNA isolation and qRT-PCR:** Total RNA extraction was performed using the FastPure Cell/Tissue Total RNA Isolation Kit (Vazyme, China). Reverse transcription was conducted with the Reverse Transcription System (Promega). Gene expression levels were determined using the 2-△△Ct method with GAPDH serving as the internal reference. The qPCR primers used were synthesized by Tsingke Biological Technology (Beijing, China) and were listed in the Supporting Information (Table S4, Supporting Information).

**Cell viability assay：** Cells (5×10^3^ per well) were seeded in 96-well plates, and cell viability was detected using the cell counting kit-8 (CCK8) assay (Dojindo Molecular Technologies, Japan) following the manufacturer’s instructions.

**Soft agar colony formation assay：**Mix 750 μL of 1.25% soft agar with 750 μL of 2×1640 complete medium, and layer it into a 6-well plate. Set overnight to form the bottom layer. The next day, mix 750 μL of 0.75% soft agar with 750 μL of 2×1640 complete medium and 7 × 10^3^ cells. Layer this mixture on top of the solidified bottom layer. Periodically supplement with 1×1640 complete medium. On day 20, fix the colonies with 4% paraformaldehyde and stain them with 1×Giemsa solution. Colonies were imaged and counted.

**Cell apoptosis and cell cycle analysis：**To assess cell apoptosis, cells were collected and stained with the Annexin V-FITC kit (BD Biosciences, USA). For cell cycle evaluation, cells were collected and fixed in 70% ice-cold ethanol overnight at 4°C. The following day, cells were treated with a propidium iodide/RNase A staining solution (Sigma-Aldrich) for 20 minutes at room temperature. Flow cytometry (Beckman Gallios, Germany) was used to analyze the samples, and cell cycle distribution was determined by FlowJo v10 software (Tree Star, USA).

**Western blot analysis：**Cellular protein lysates were prepared using RIPA buffer supplemented with 1 mM PMSF and 1% phosphatase inhibitor cocktail. Proteins (20 μg) were resolved on SDS-PAGE and transferred to PVDF membranes. For western blotting, the membranes were examined with antibodies against GAPDH (MAB374, Millipore), IRF2BP2 (HPAO27815, Sigma-Aldrich), PARP (#9542, CST), c-Myc (A19032, Abclonal), BRD4 (#13440, CST), Caspase-8 (#9746, CST), CDK1 (19532-1-AP, Proteintech), CDK2 (AF1063, Beyotime), CDK4 (12790, Cell Signaling Technology), Cyclin D1 (60186-1-Ig, Proteintech), RUNX1 (ab272456, Abcam), ERG (14356-1-AP, proteintech), ETS1 (66598-1-Ig, Proteintech), ELF1 (ab64937, Abcam)，RAG1 (#3968S, CST). Detection of antibody-antigen interactions was achieved using a chemiluminescent HRP substrate (Millipore, USA). All antibodies were listed in the Supporting Information (Table S5, Supporting Information)

**RNA-seq, Cleavage Under Targets & Tagmentation (CUT&Tag), and data analysis:** RNA sequencing was conducted by Novogene Bioinformatics Technology Co., Ltd. (Beijing, China), encompassing RNA isolation, library preparation, transcriptome sequencing using Illumina NovaSeq 6000, and clean data filtering. The resulting 150 bp paired-end reads were aligned to the hg38 genome (Ensembl) employing HISAT2 software (version 2.2.0). StringTie software (version 2.1.2) was utilized for transcriptome assembly and abundance analysis. Differentially expressed genes were detected using the R/Bioconductor package DESeq2. Gene set enrichment analysis (GSEA) was performed using the R/Bioconductor package clusterProfiler, utilizing Hallmarks gene sets sourced from the Molecular Signatures Database (MSigDB).

CUT&Tag assays were conducted using the Hyperactive Universal CUT&Tag Assay Kit for Illumina (TD903, Vazyme) following the manufacturer's instructions. DNA was extracted and amplified with i5 and i7 primers in TruePrep Index Kit V2 for Illumina (#TD202, Vazyme). Libraries were purified with VAHTS DNA Clean Beads (#N411, Vazyme) and sequenced by Novogene Bioinformatics Technology Co., Ltd (Beijing, China). The 150 bp paired-end reads were aligned to the hg38 (Ensembl) genome using bowtie2 (version 2.4.4) with parameters --end-to-end --verysensitive --no-mixed --no-discordant. Duplicates were removed using Picard tools. Peaks were called using MACS (version 3.0).

**Supplementary data**

**Figure S1.** Hockey stick plots show super-enhancer genes(including IRF2BP2) in 6 T-ALL cell lines and 7 T-ALLpatients .

**Figure S2.** Visualization of the ChIP-seq data on chr1:234599841-234612606 identified candidate genes A) CDK6, B) ELF1.

**Figure S3.** Visualization of the ChIP-seq data on chr1:234599841-234612606 identified candidate genes A) ELMSAN1, B) ELOVL5.

**Figure S4.** Visualization of the ChIP-seq data on chr1:234599841-234612606 identified candidate genes A) MSI2, B) ALM2AKAP2P.

**Figure S5.** Visualization of the ChIP-seq data on chr1:234599841-234612606 identified candidate genes A) SPTBN1. B) CDK6, ELF1, ELMSAN, ELOVL5, MSI2, PALM2AKAP2 and SPTBN1 mRNA expression in T-ALL patient samples (N=18) compared to normal T cells (N=4). two-tailed unpaired *t* test in panel B; data were shown as mean±s.e.m.

**Figure S6.** IRF2BP2 protein levels in T-ALL cell lines compared to CD3T cells.

**Figure S7.** Genomic distribution of ELF1, ERG, ETS1 and RUNX1 in J.gamma1 cells.

**Figure S8.** Knockdown IRF2BP2 in Jurkat and J.gamma1 cells A) Cell cycle analysis showed that IRF2BP2 knockdown led to an extension of the G2 phase. B-C) Soft agar colony formation assays (N=3) showed a reduced number of colonies in IRF2BP2-knockdown cells compared to the control group. two-tailed unpaired *t* test in panel C; data were shown as mean±s.e.m.

**Figure S9.** Using the CRISPR/Cas9 system to knockout IRF2BP2 in Jurkat and J.gamma1 cell lines A) Knockout IRF2BP2 cells exhibited membrane shrinkage, morphological changes, and an increased presence of debris in the culture medium. B) IRF2BP2 mRNA expression after IRF2BP2 knockout (N=3). C) CCK8 of knockout IRF2BP2 led to inhibited cell proliferation (N=3). D,E) Flow cytometry analysis revealed a significant increase in apoptosis after IRF2BP2 knockout (N=3). F) Western blot analysis indicated that IRF2BP2 knock out resulted in decreased expression of IRF2BP2, CDK4 and BCL2. two-tailed unpaired *t* test in panel B and E; two-way ANOVA test for analysis in panel C; data were shown as mean±s.e.m.

**Figure S10.** H.E. staining and immunohistochemical analysis for IRF2BP2, Ki67 and c-Myc demonstrated reduced tumor infiltration in the liver, spleen, and bone marrow following IRF2BP2 knockdown (N=3). two-tailed unpaired *t* test in panel E; data were shown as mean±s.e.m.

**Figure S11.** Successfully constructed knockout Vav-iCRE^+/-^;IRF2BP2^fl/fl^ mice. A) Schematic diagram illustrating the primer design for the identification of gene knockout mice. B) Primer sequences for the identification of gene knockout mice. C)Primer sequences for the identification of Vav-icre mice. D)Analysis of electrophoresis results and PCR identification of gene knockout mice

**Figure S12.** CD25+ Treg cells in the peripheral blood, spleen, and bone marrow followingin Vav-iCRE^+/-^;IRF2BP2^fl/fl^ mice (N=3). two-tailed unpaired *t* test in Figure S12; data were shown as mean±s.e.m.

**Figure S13.** Magnetically sorted CD3^+^ T cells from the spleens, A) Mode diagram of CD3+ T cells from the spleens of Vav-iCre+/-;Irf2bp2fl/fl mice. B) Flow cytometry test CD3^+^T cells from the spleens of *Vav-iCre^+/-^;Irf2bp2^fl/fl^* mice and *Vav-iCre^-/-^;Irf2bp2^fl/fl^* controls.

**Figure S14.** T cells from Vav-iCRE^+/-^;IRF2BP2^fl/fl^ mice affectsnE2F and STAT5 pathway. The RNA-seq analysis on T cells from *Vav-iCre^+/-^;Irf2bp2^fl/fl^* mice versus control. B-C) Gene Set Enrichment Analysis (GSEA) using Hallmark gene sets revealed significant enrichment for E2F-target genes and STAT5-target genes in the *Vav-iCre^+/-^;Irf2bp2^fl/fl^* mice compared to controls.

**Figure S15.** Gene Set Enrichment Analysis (GSEA) using Hallmark gene sets revealed significant enrichment for E2F-target genes and MYC-target genes in the IRF2BP2-knockdown group compared to controls.

**Figure S16.** CUT&Tag analysis to determine the targets of IRF2BP2 in J.gamma1 cells.

**Figure S17** RUNX1, ERG, and ELF1 are SE-driven genes that are highly expressed in T-ALL cells.

**Figure S18.** Western blot of RUNX1, ELF1, ERG, MYCN, MEIS2 and HAND2 in T-ALL cell lines compared to neuroblastoma cells.

**Figure S19** Knockdown RAG1 in Jurkat cells. A-B) Knockdown IRF2BP2 resulted in decreased RAG1 expression in Jurkat cells (N=3). C) Knockdown RAG1 led to inhibited growth (N=3), D-E) The mRNA and protein expression of Knockdown RAG1 in Jurkat cells (N=3), F-G) Apoptosis of knockdown RAG1 in Jurkat cells (N=3). two-tailed unpaired *t* test in panel B, D and G; two-way ANOVA test for analysis in C; data were shown as mean±s.e.m.

**Figure S20. RAG2 sustains the growth and survival of T-ALL cells.** A-B) Knockdown IRF2BP2 result in decreased RAG2 expression in Jurkat and J.gamma1 cells (N=3). C-D) Knockdown RAG2 led to inhibited growth (N=3), E) The protein expression of RAG2 of Knockdown RAG2 in Jurkat and J.gamma1 cells, two-tailed unpaired *t* test in panel B; two-way ANOVA test for analysis in D; data were shown as mean±s.e.m.

**Table S1.** Information of seven T-ALL patients.

**Table S2.** Landscape and annotation of super-enhancers in T-ALL patients.

**Table S3.** Landscape and annotation of super-enhancers in T-ALL cell lines.

**Table S4.** Primers and antibodies used in the manuscript.

**Table S5.** RNA-seq results of CD3+ T cells from the spleens of Vav-iCre+/-; Irf2bp2fl/fl mice versus control.

**Table S6.** GSEA analysis results with Hallmark signatures for Irf2bp2-knockout CD3+ T cells versus control.

**Table S7.** RNA-seq results of IRF2BP2-knockdown J.gamma1 cells vesus control.

**Table S8.** GSEA analysis results with Hallmark signatures for IRF2BP2-knockdown J.gamma1 cells versus control.

**Table S9.** Binding profiles of IRF2BP2 in J.gamma1 cells identified by CUT&Tag.

**Table S10.** Binding profiles of RUNX1 in J.gamma1 cells identified by CUT&Tag.

**Table S11.** Binding profiles of ELF1 in J.gamma1 cells identified by CUT&Tag.

**Table S12.** Binding profiles of ERG in J.gamma1 cells identified by CUT&Tag

**Table S13.** Binding profiles of ETS1 in J.gamma1 cells identified by CUT&Tag

**Table S14.** EnrichR analysis for overlap of IRF2BP2-binding genes and IRF2BP2-upregulated genes

**Table S15.** EnrichR analysis for overlap of IRF2BP2-binding genes and IRF2BP2-downregulated genes.

**Supplementary file 1.** STR analysis of cell lines.

**Supplementary file 2.** Full uncropped Western Blots image(s).

**Figure S1**


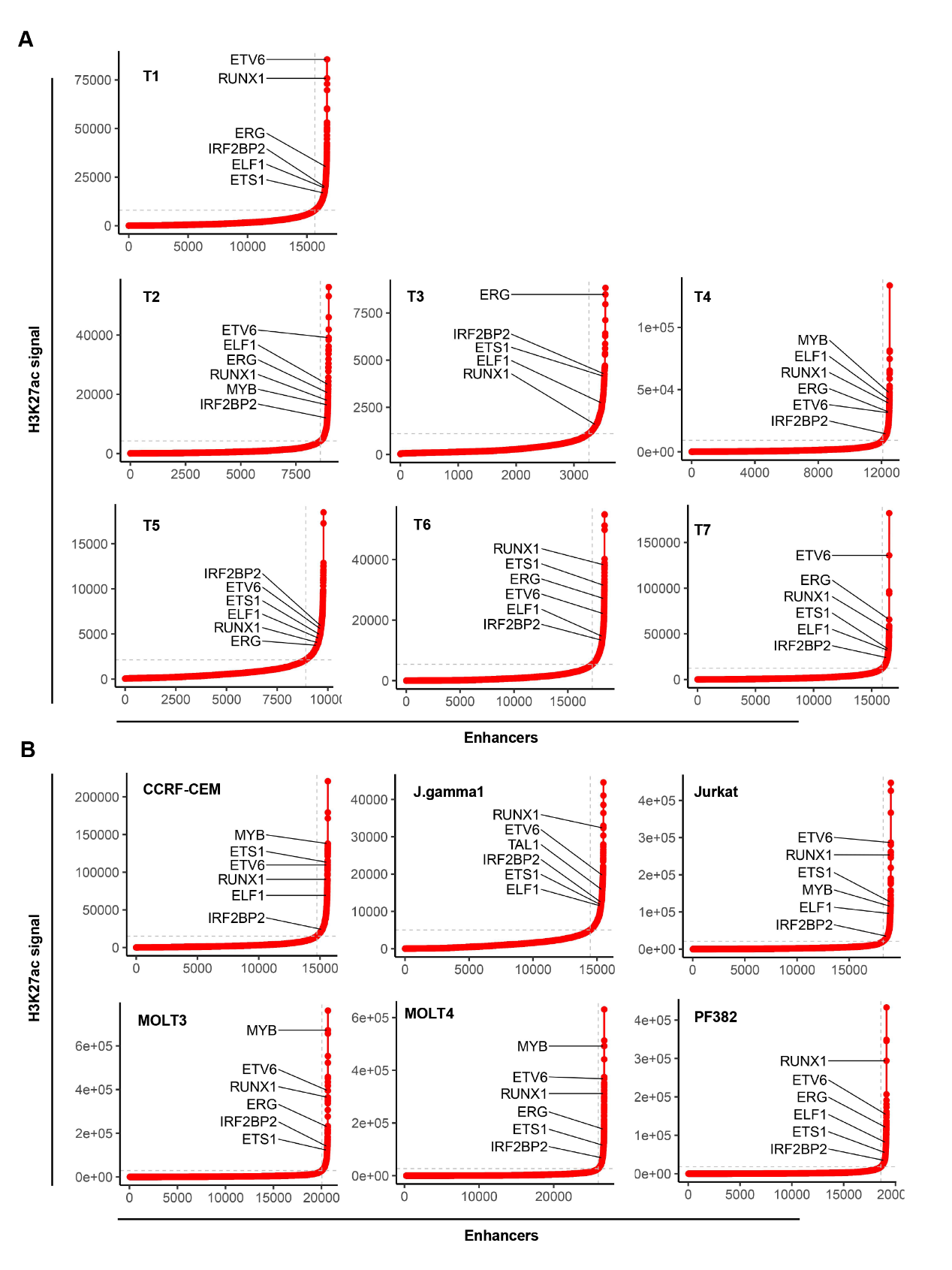


**Figure S2**


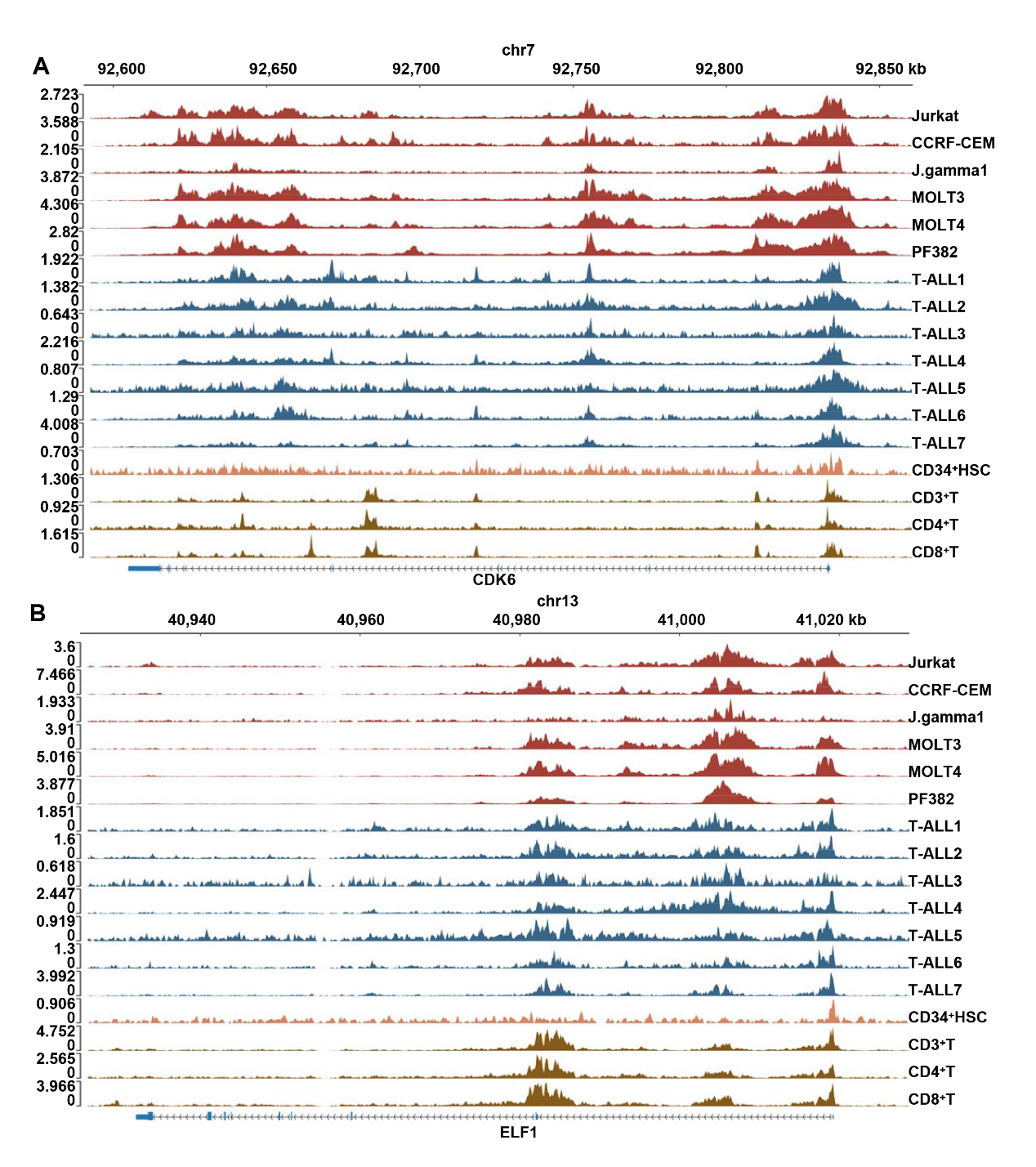


**Figure S3**


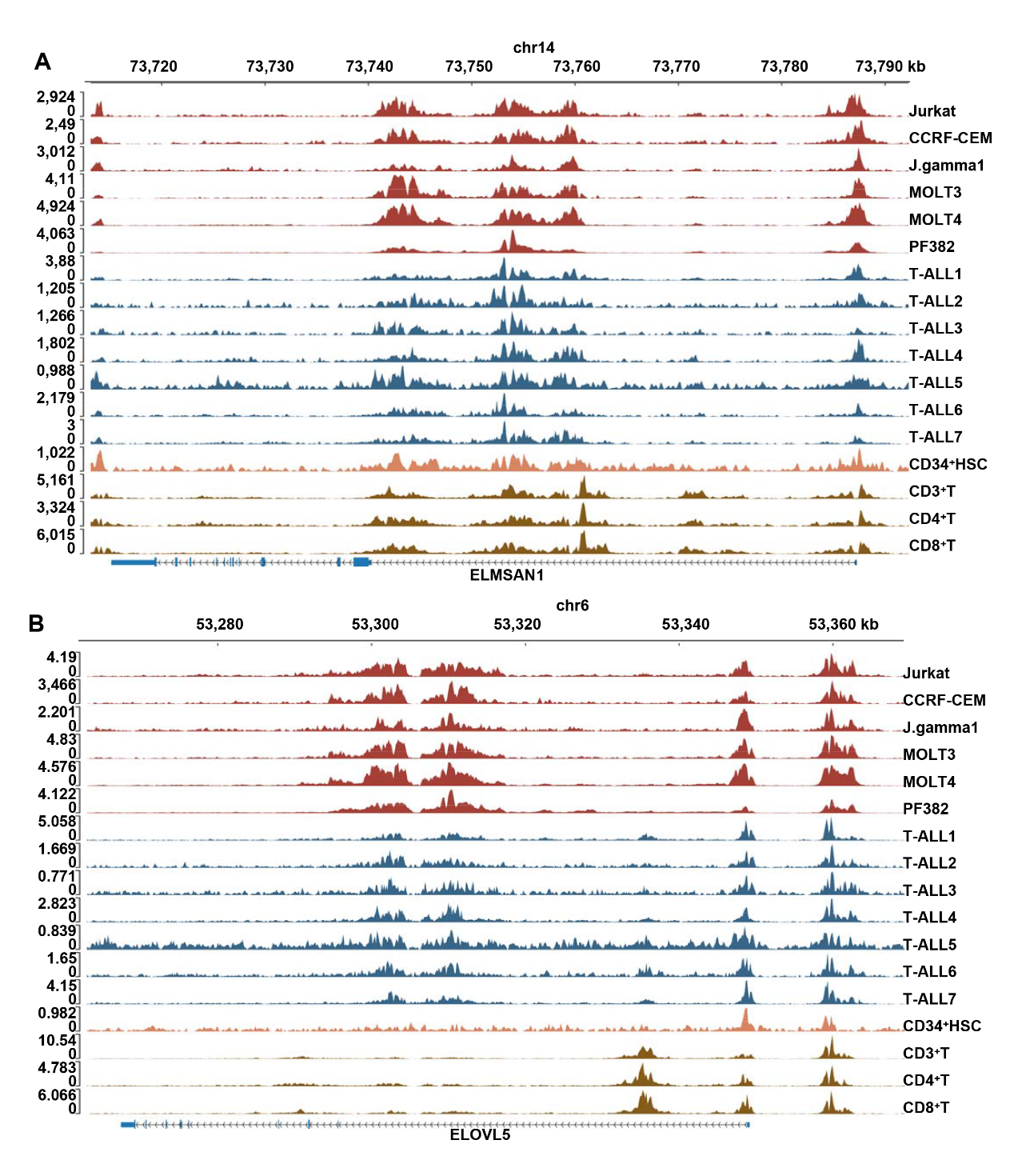


**Figure S4**


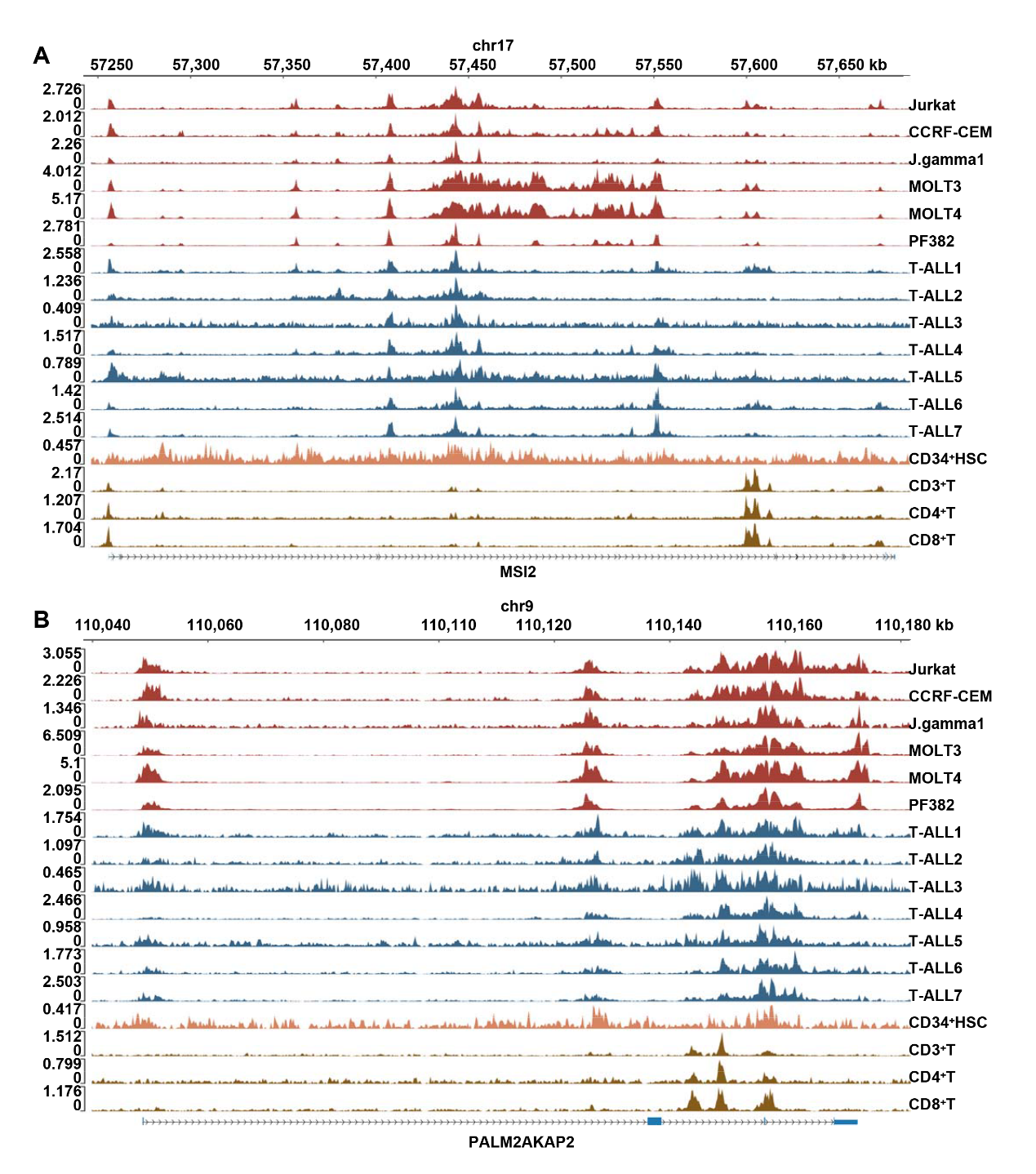


**Figure S5**


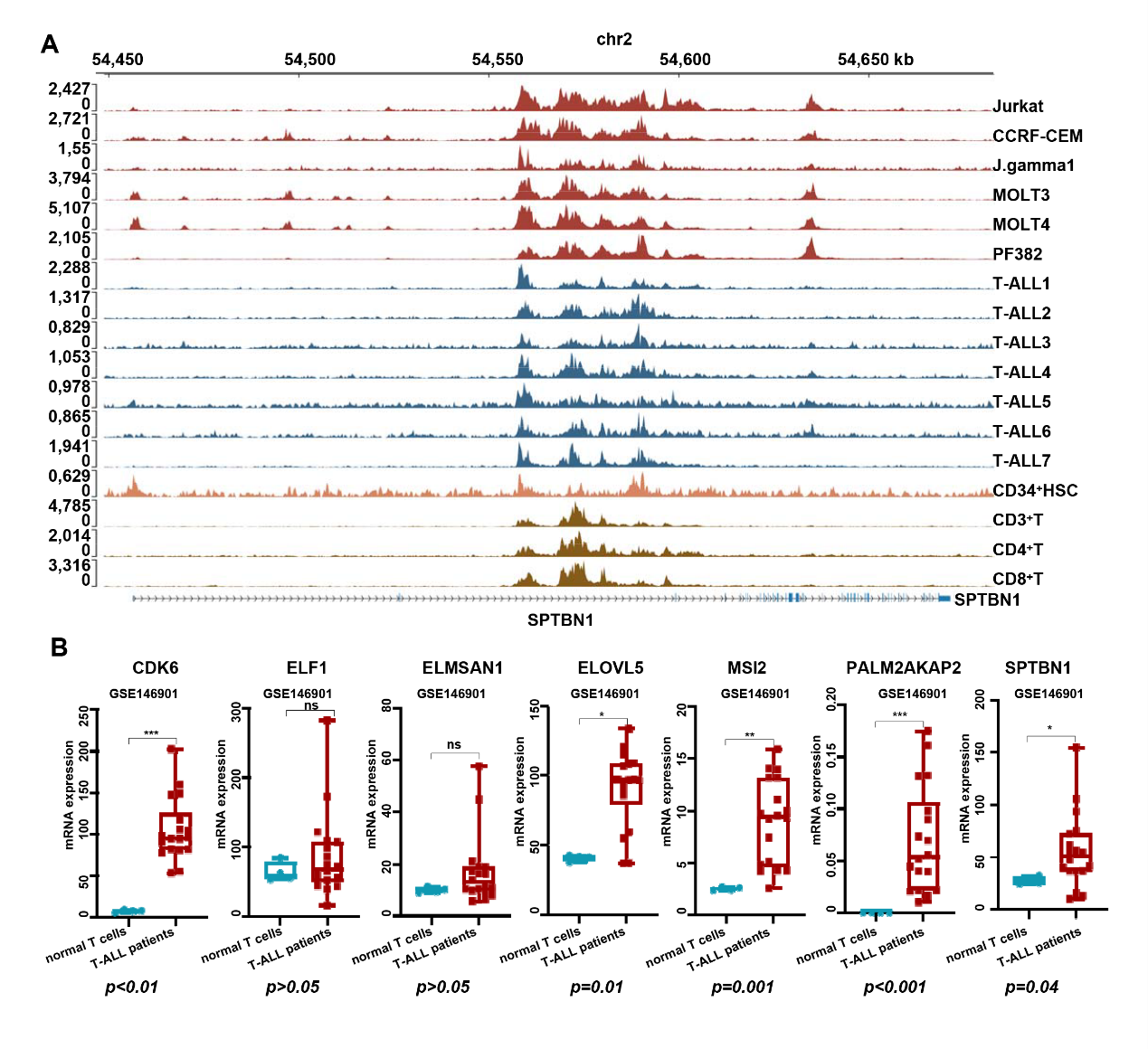


**Figure S6**


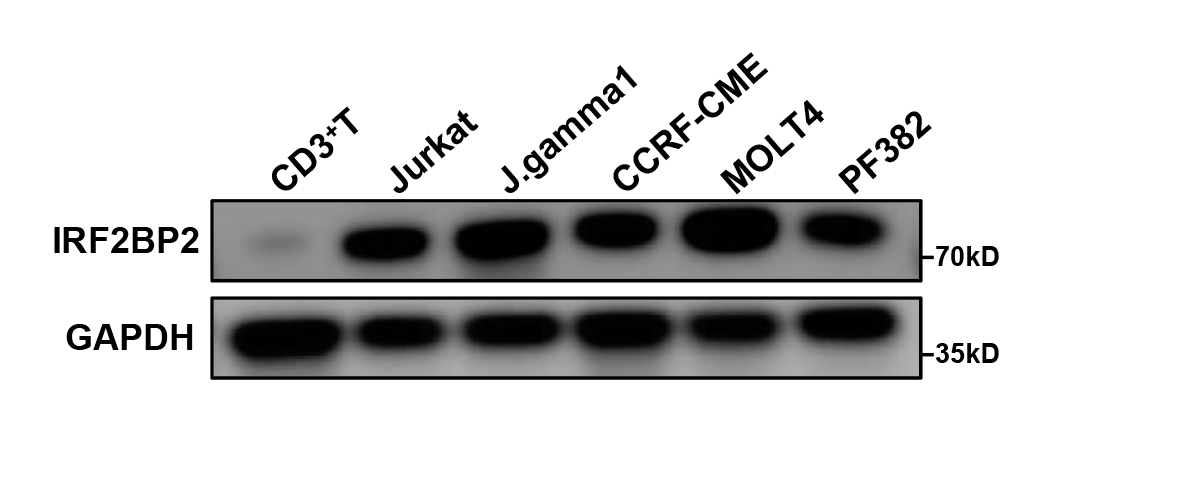


**Figure S7**


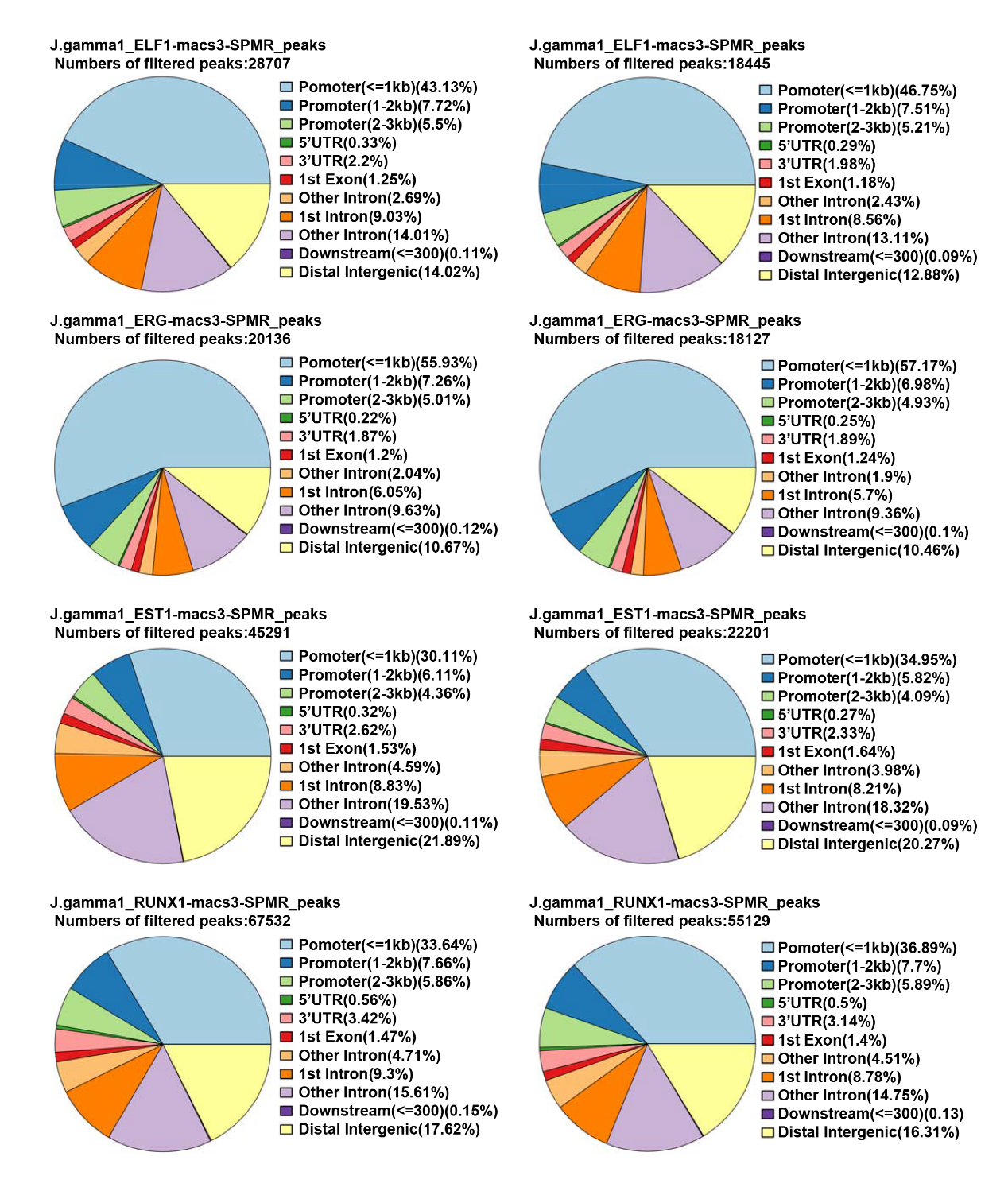


**Figure S8**


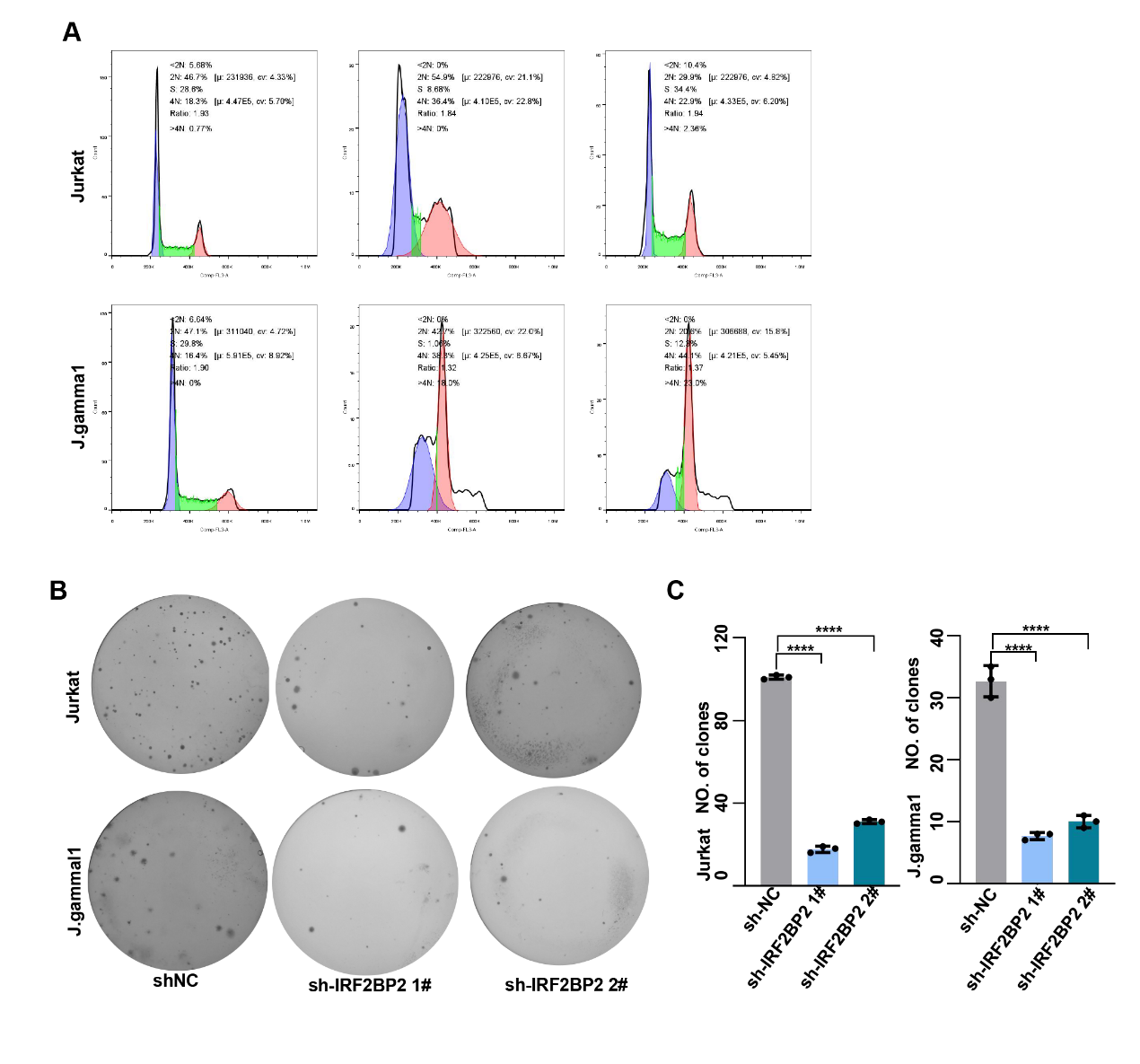


**Figure S9**


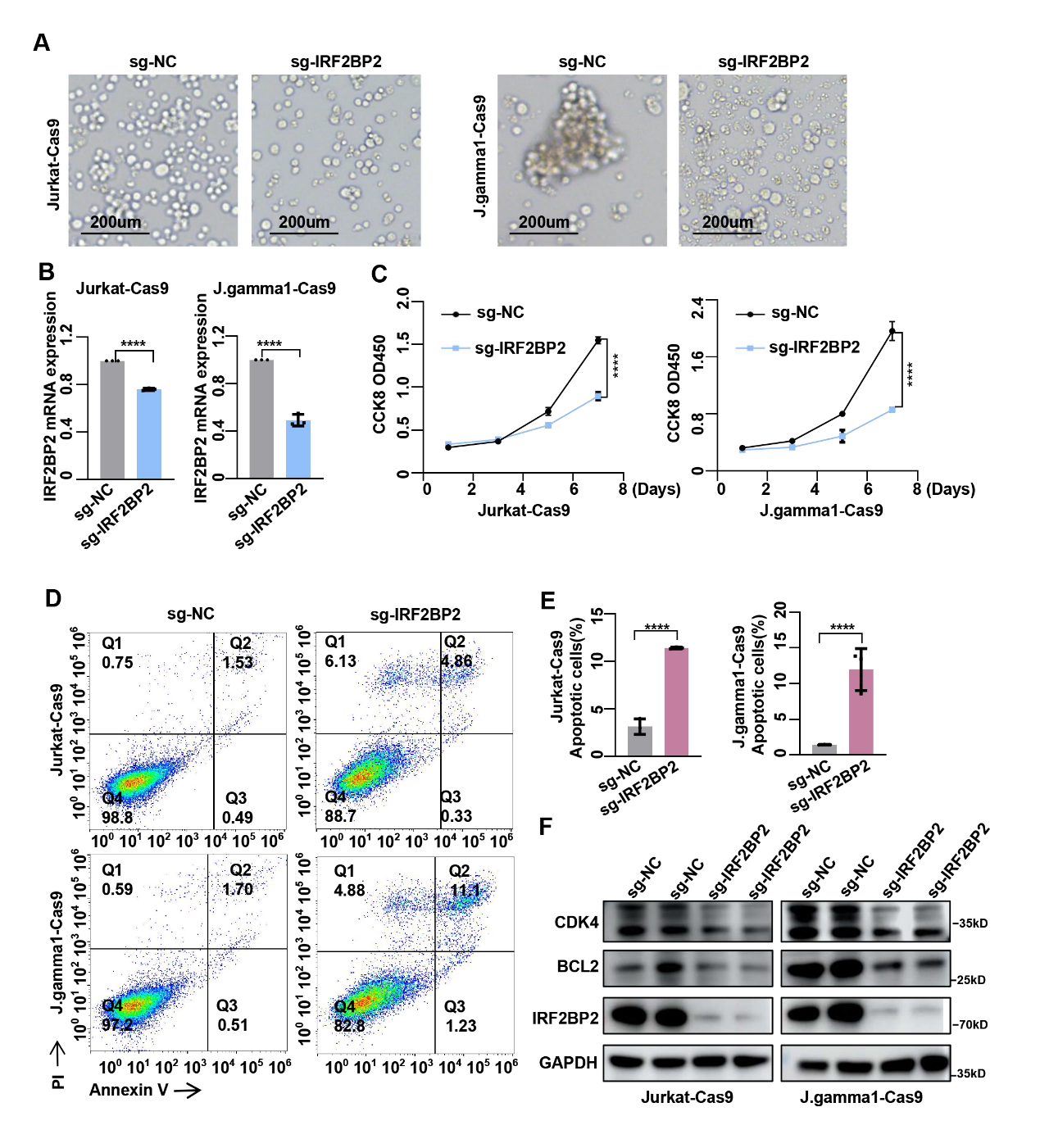


**Figure S10**


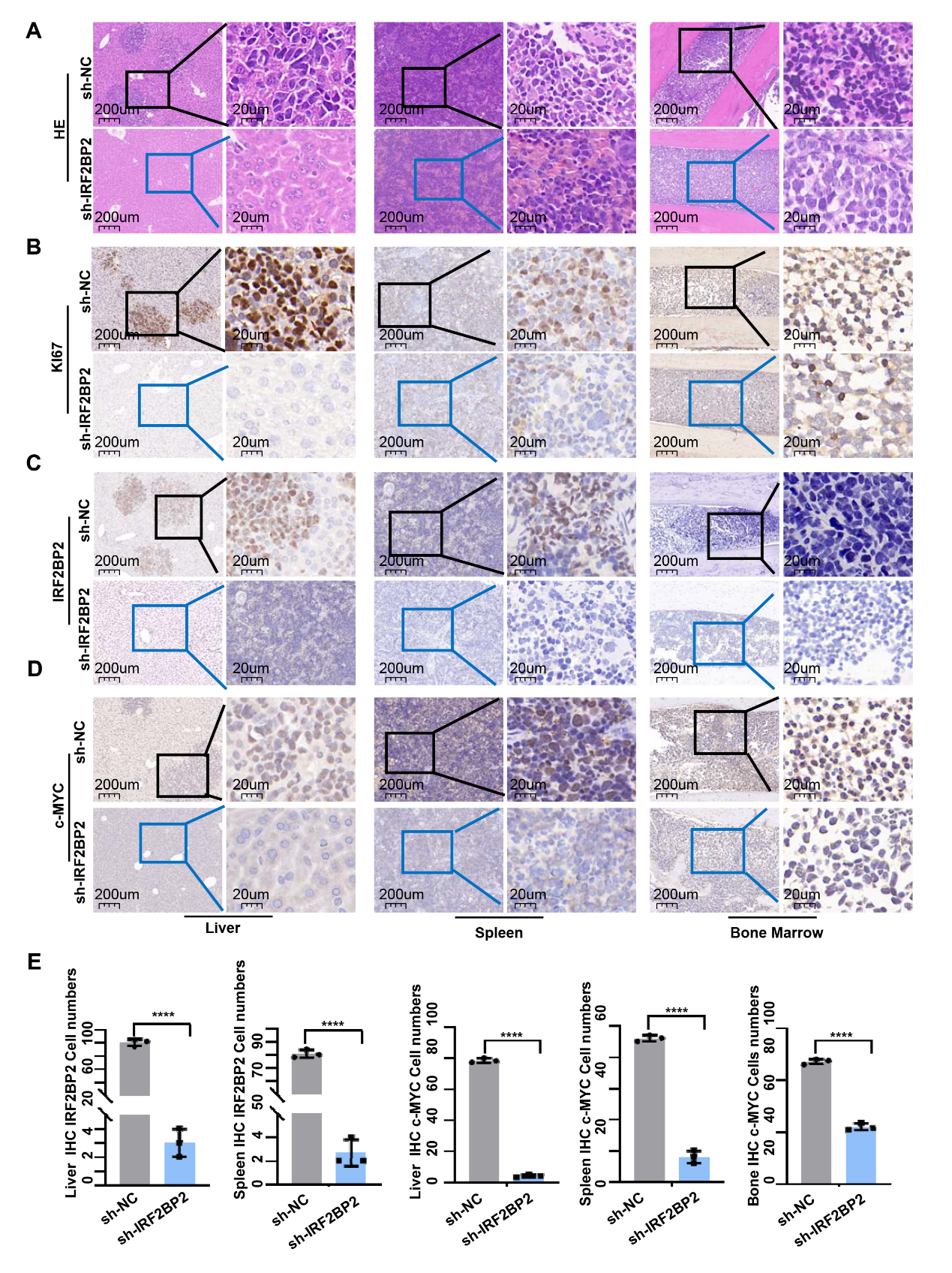


**Figure S11**


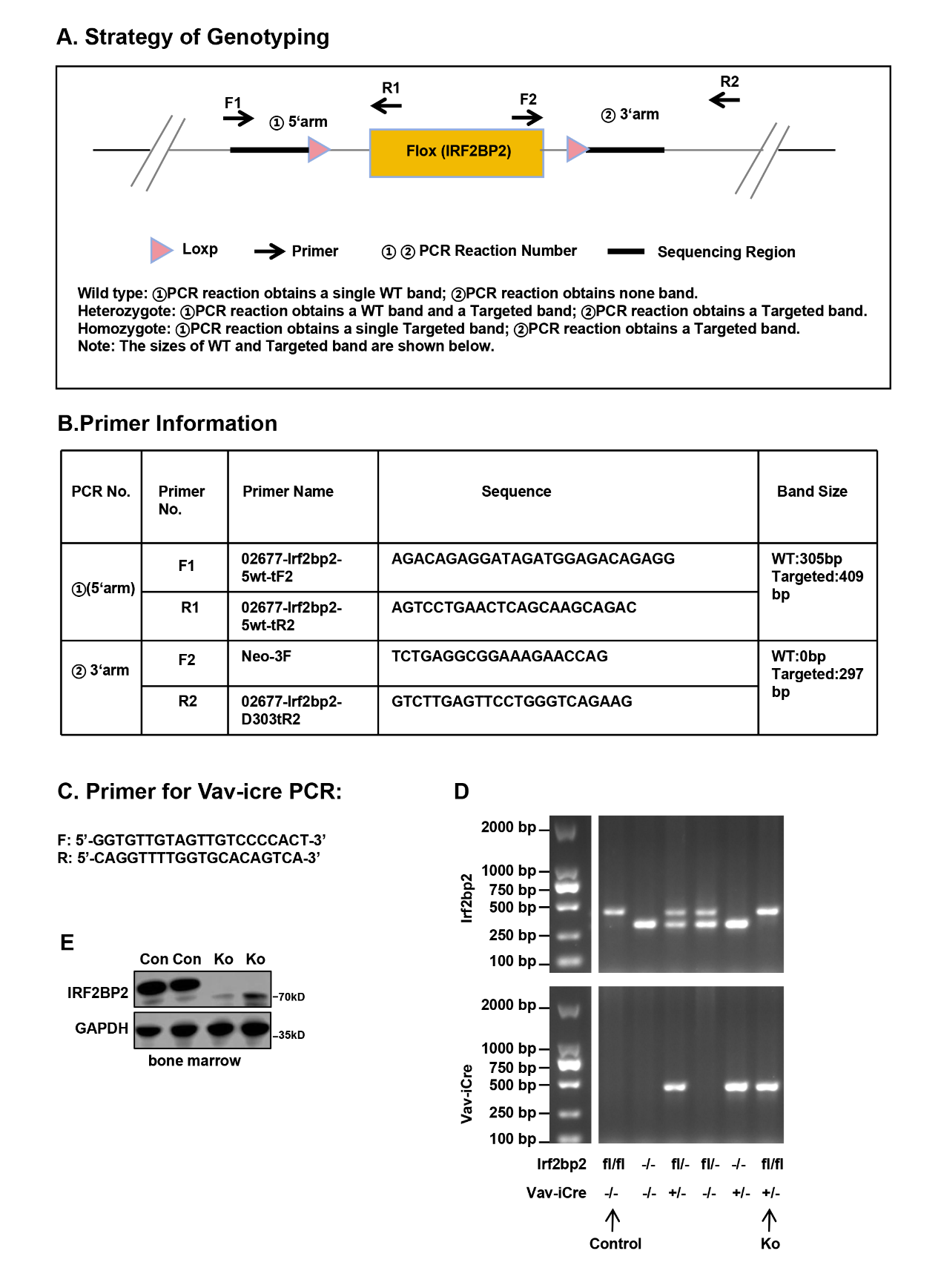


**Figure S12**


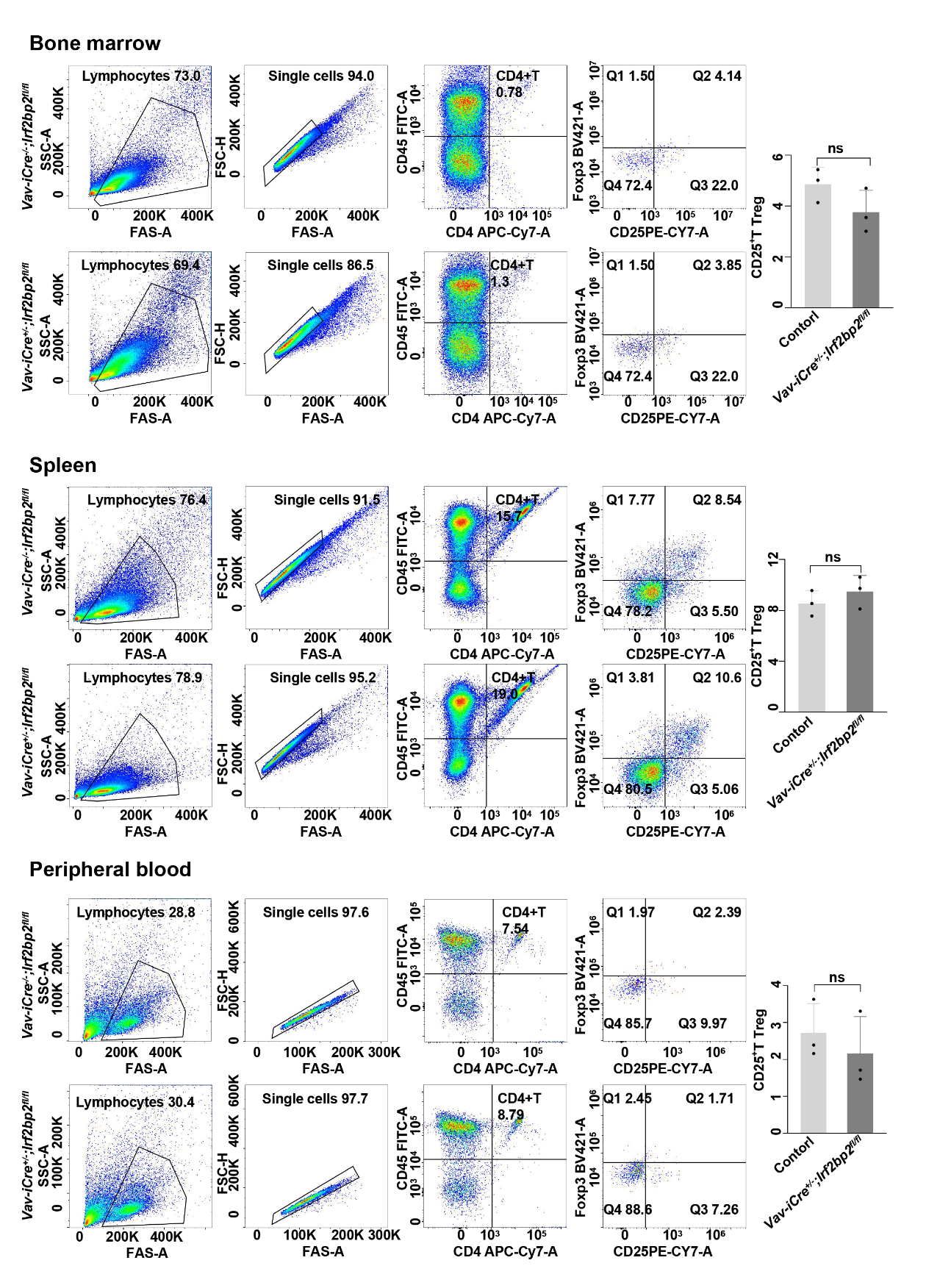


**Figure S13**


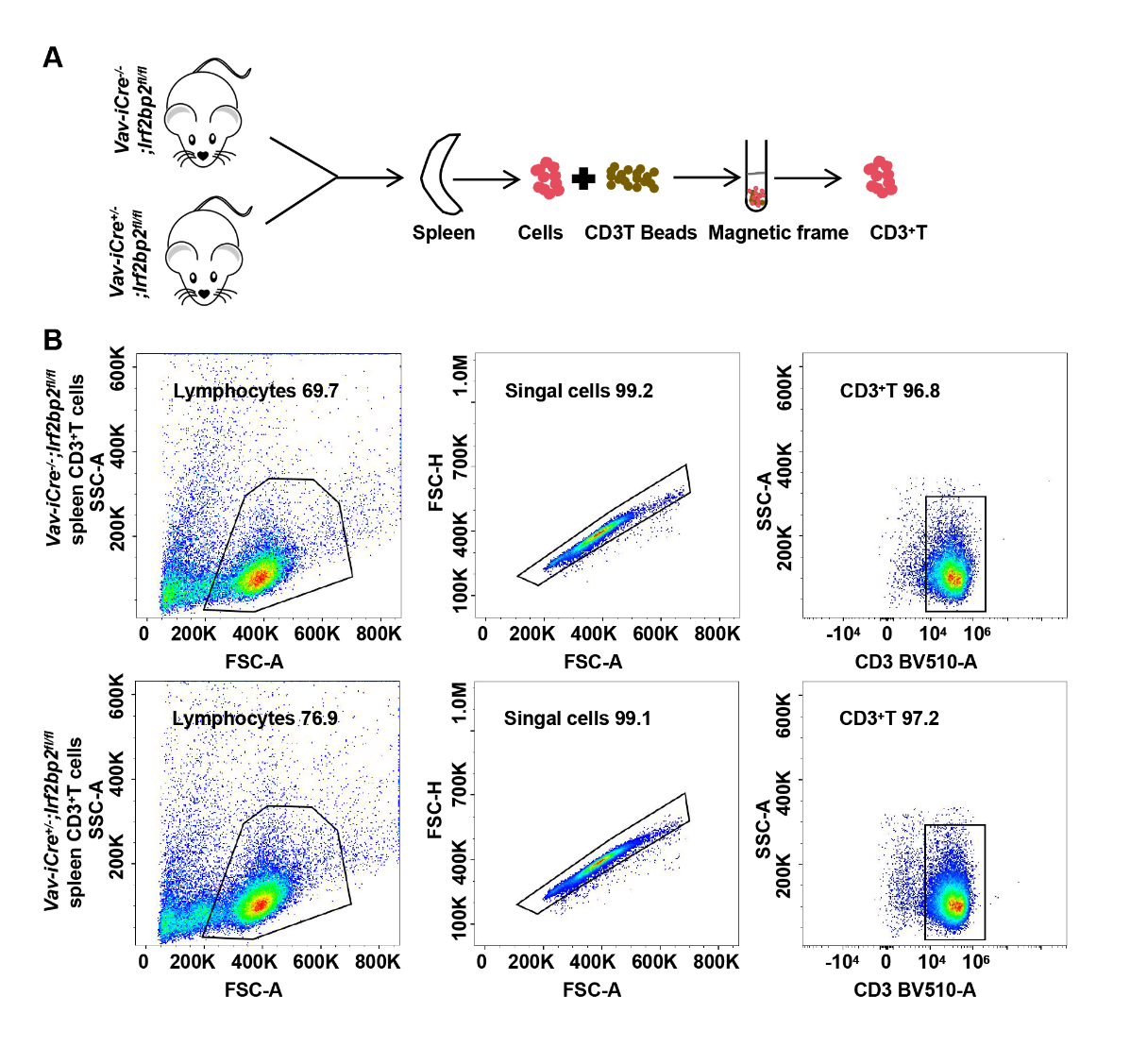


**Figure S14**


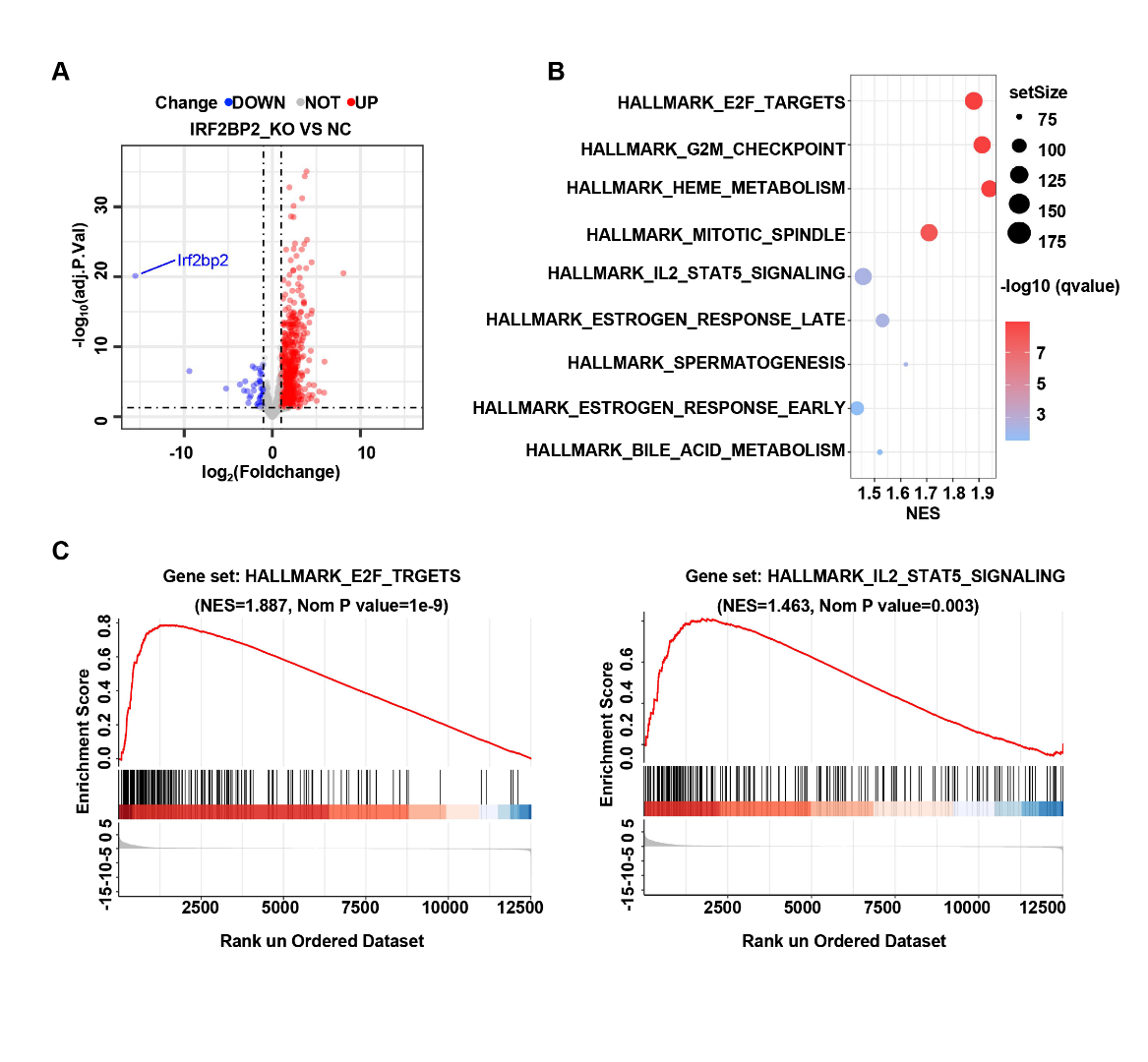


**Figure S15**


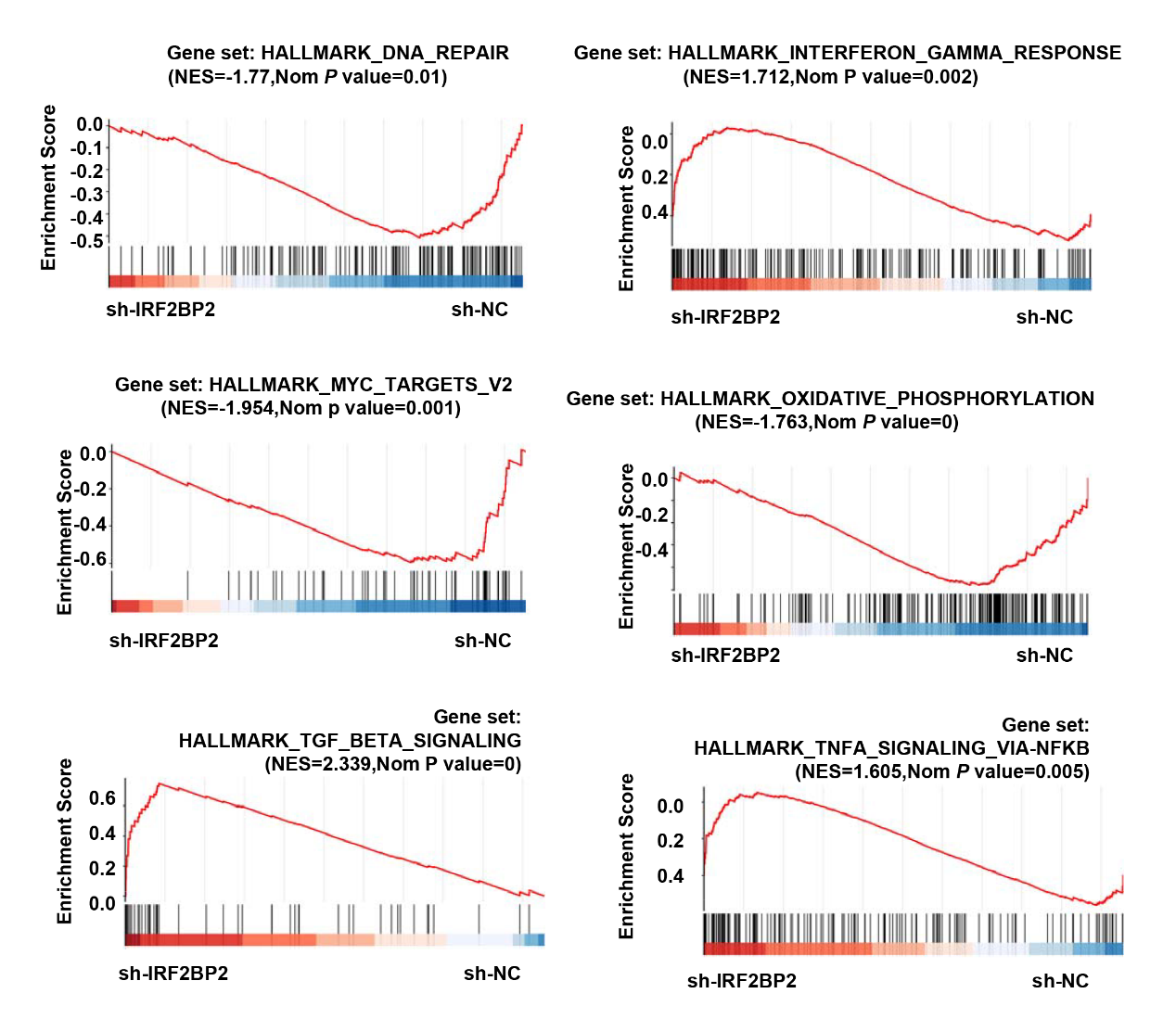


**Figure S16**

**
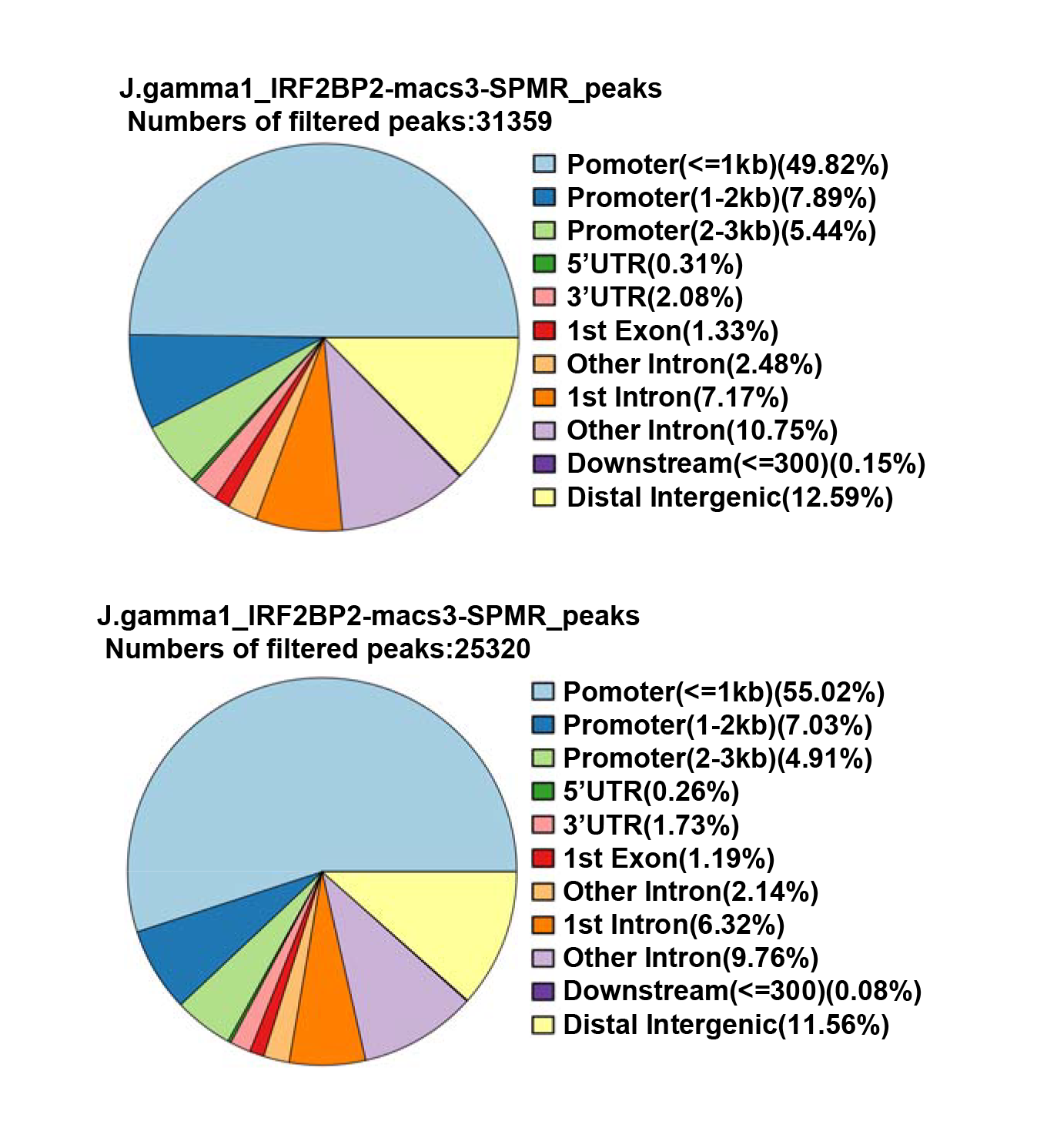
**

**Figure S17**

**
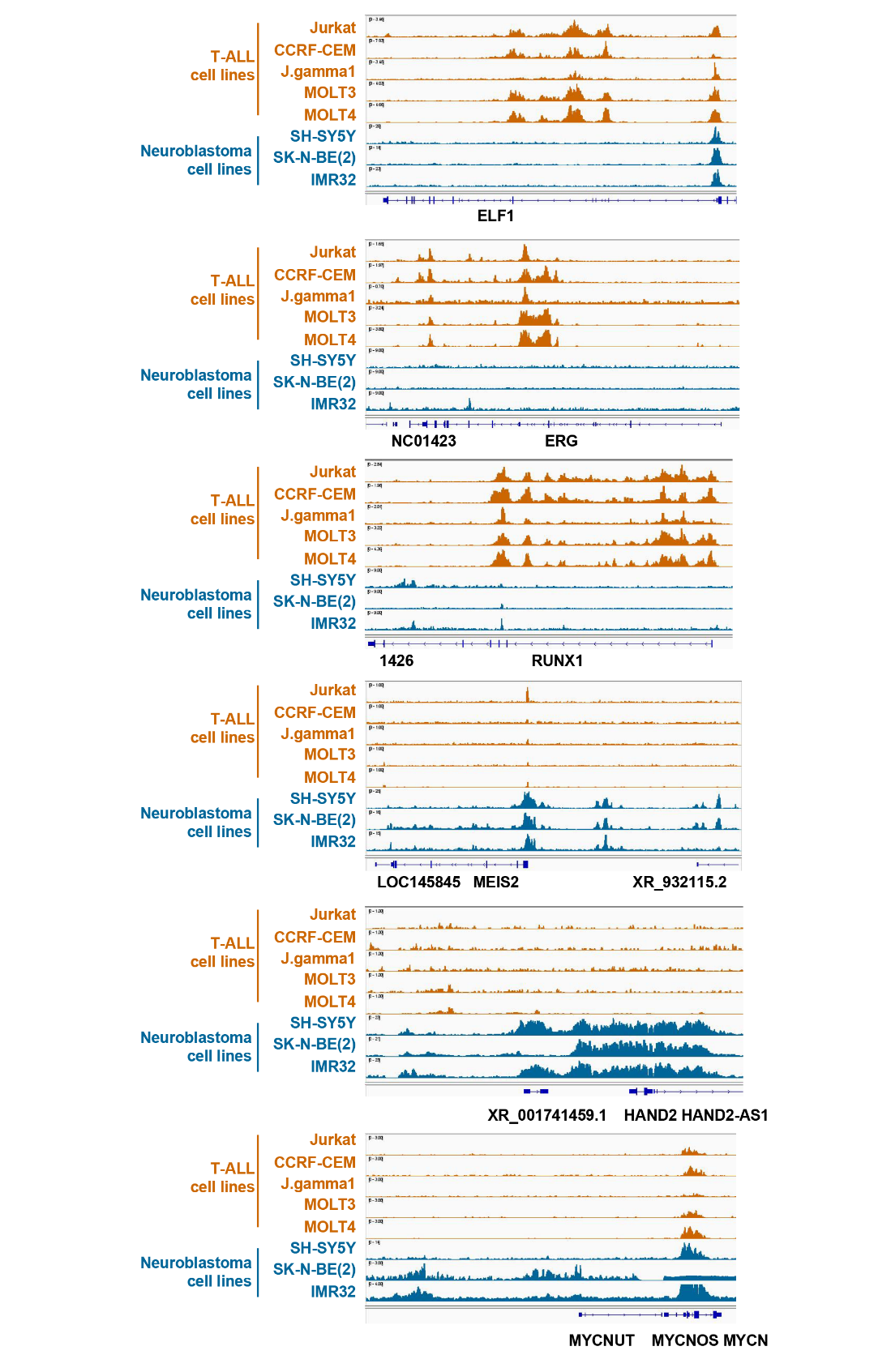
**

**Figure S18**


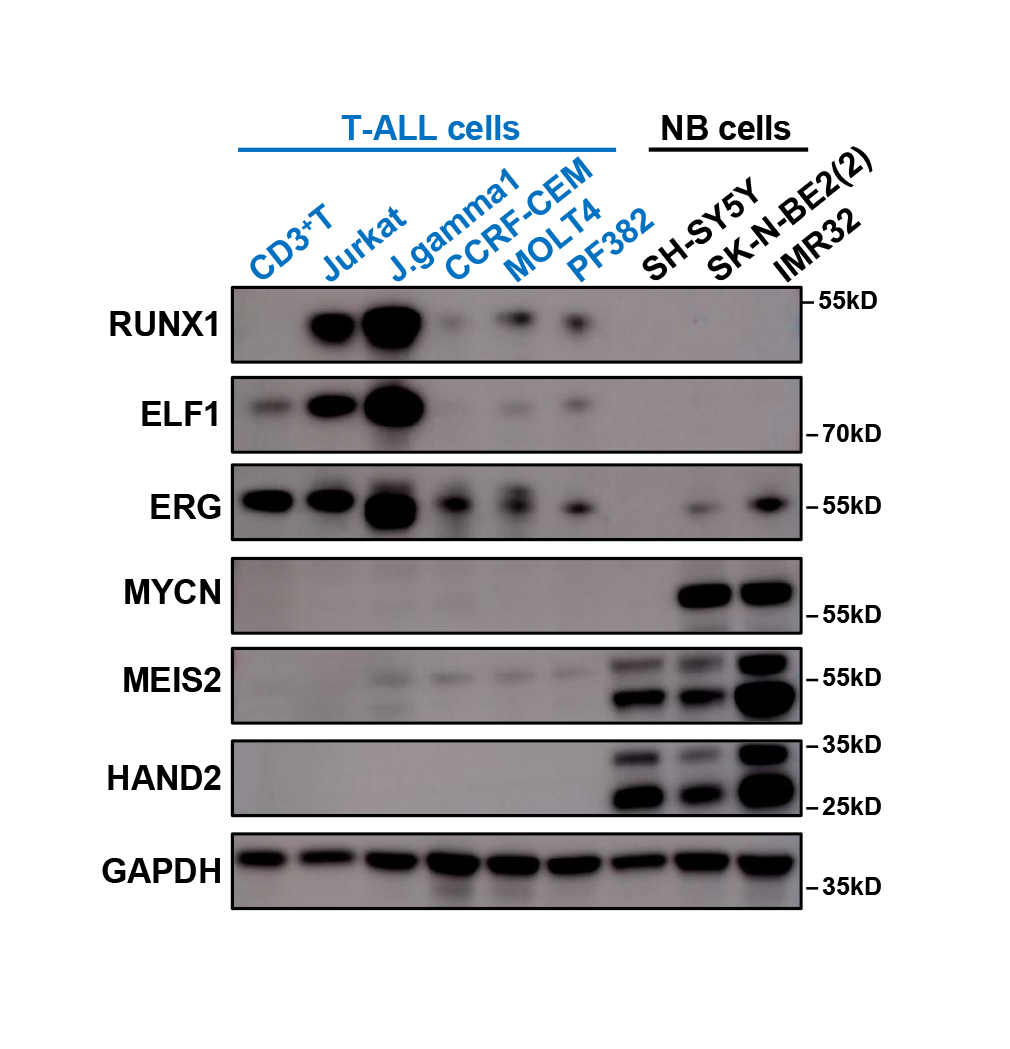


**Figure S19**


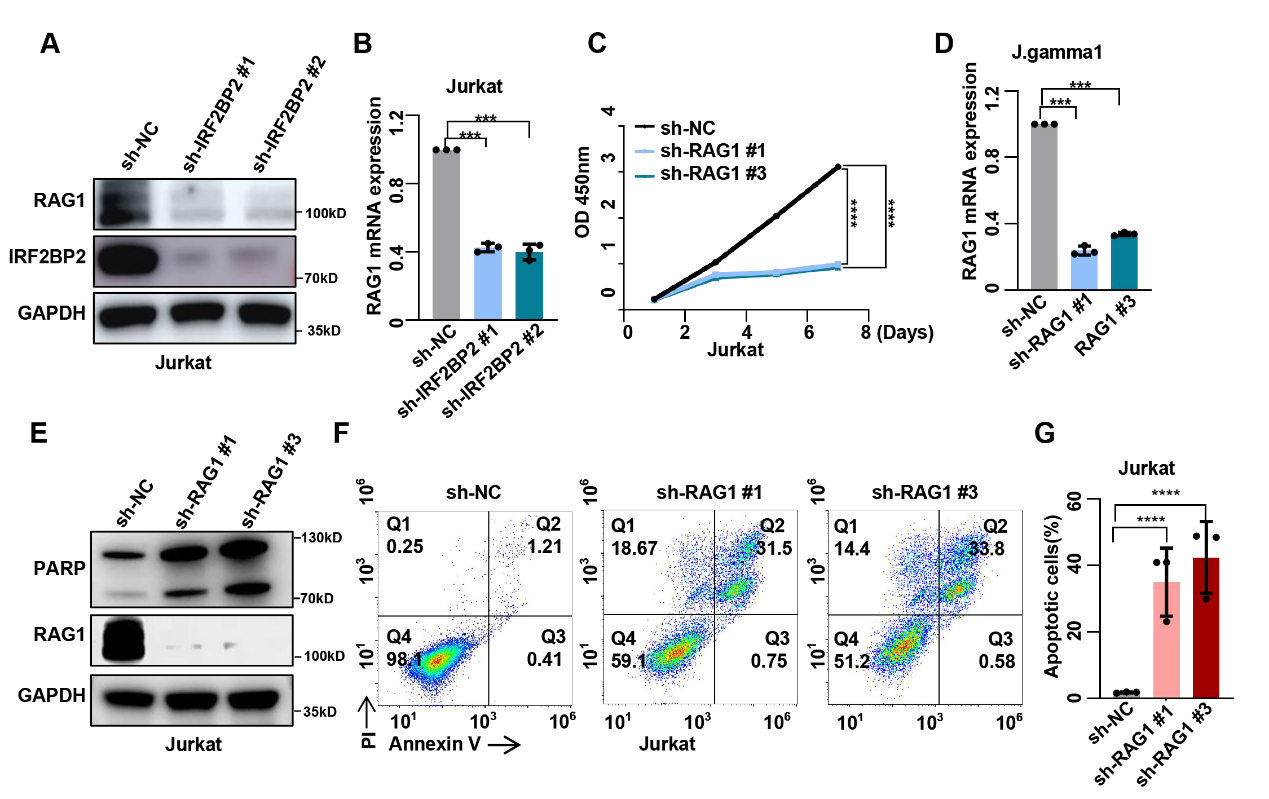


**Figure S20**


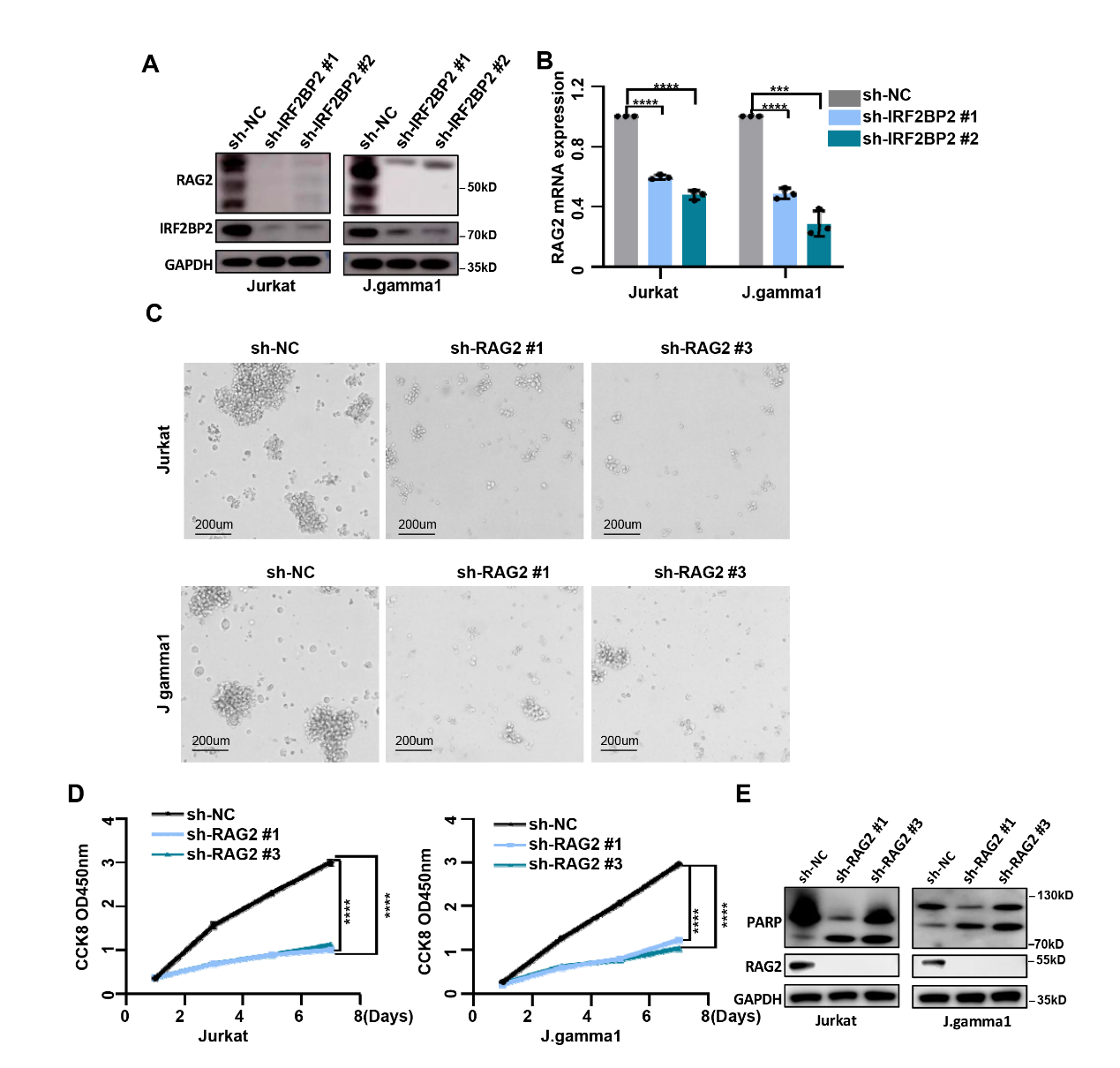

Supplement: Supplementary file 1 — Supporting Information [file ADVS-12-2407113-s001.docx]
